# Serum IL-6, sAXL, and YKL-40 as systemic correlates of reduced brain structure and function in Alzheimer’s disease: results from the DELCODE study

**DOI:** 10.1186/s13195-022-01118-0

**Published:** 2023-01-12

**Authors:** Frederic Brosseron, Anne Maass, Luca Kleineidam, Kishore Aravind Ravichandran, Carl-Christian Kolbe, Steffen Wolfsgruber, Francesco Santarelli, Lisa M. Häsler, Róisín McManus, Christina Ising, Sandra Röske, Oliver Peters, Nicoleta-Carmen Cosma, Luisa-Sophie Schneider, Xiao Wang, Josef Priller, Eike J. Spruth, Slawek Altenstein, Anja Schneider, Klaus Fliessbach, Jens Wiltfang, Björn H. Schott, Katharina Buerger, Daniel Janowitz, Martin Dichgans, Robert Perneczky, Boris-Stephan Rauchmann, Stefan Teipel, Ingo Kilimann, Doreen Görß, Christoph Laske, Matthias H. Munk, Emrah Düzel, Renat Yakupow, Laura Dobisch, Coraline D. Metzger, Wenzel Glanz, Michael Ewers, Peter Dechent, John Dylan Haynes, Klaus Scheffler, Nina Roy, Ayda Rostamzadeh, Annika Spottke, Alfredo Ramirez, David Mengel, Matthis Synofzik, Mathias Jucker, Eicke Latz, Frank Jessen, Michael Wagner, Michael T. Heneka, the DELCODE study group

**Affiliations:** 1 German Center for Neurodegenerative Diseases (DZNE), Venusberg-Campus 1, 53127 Bonn, Germany; 2 Department of Neurodegenerative Disease and Geriatric Psychiatry, University of Bonn Medical Center, Venusberg-Campus 1, 53127 Bonn, Germany; 3 German Center for Neurodegenerative Diseases (DZNE), Leipziger Straße 44, 39120 Magdeburg, Germany; 4 Institute of Innate Immunity, University of Bonn Medical Center, Venusberg-Campus 1, 53127 Bonn, Germany; 5 Bayer AG, Alfred-Nobel-Straße 50, 40789 Monheim am Rhein, Germany; 6 Hertie Institute for Clinical Brain Research, Department Cellular Neurology, University of Tübingen, Otfried-Müller-Strasse 27, 72076 Tübingen, Germany; 7 German Center for Neurodegenerative Diseases (DZNE), Otfried-Müller-Straße 27, 72076 Tübingen, Germany; 8 Excellence Cluster on Cellular Stress Responses in Aging-Associated Diseases (CECAD), University of Cologne, Joseph-Stelzmann-Strasse 26, 50931 Köln, Germany; 9 German Center for Neurodegenerative Diseases (DZNE), Charitéplatz 1, 10117 Berlin, Germany; 10 Department of Psychiatry and Psychotherapy, Charité, Charitéplatz 1, 10117 Berlin, Germany; 11 Department of Psychiatry and Psychotherapy, Charité – Universitätsmedizin Berlin, Campus Benjamin Franklin, Hindenburgdamm 30, 12203 Berlin, Germany; 12 Department of Psychiatry and Psychotherapy, Technical University Munich, 81675 Munich, Germany; 13 German Center for Neurodegenerative Diseases (DZNE), Von-Siebold-Str. 3a, 37075 Göttingen, Germany; 14 Department of Psychiatry and Psychotherapy, University Medical Center Göttingen, University of Göttingen, Von-Siebold-Str. 5, 37075 Göttingen, Germany; 15 Neurosciences and Signaling Group, Institute of Biomedicine (iBiMED), Department of Medical Sciences, University of Aveiro, Aveiro, Portugal; 16 Leibniz Institute for Neurobiology, Brenneckestr. 6, 39118 Magdeburg, Germany; 17 German Center for Neurodegenerative Diseases (DZNE), Feodor-Lynen-Strasse 17, 81377 Munich, Germany; 18 Institute for Stroke and Dementia Research (ISD), University Hospital, LMU Munich, Feodor-Lynen-Strasse 17, 81377 Munich, Germany; 19 Department of Psychiatry and Psychotherapy, University Hospital, LMU Munich, Munich, Germany; 20 Munich Cluster for Systems Neurology (SyNergy) Munich, Munich, Germany; 21 Ageing Epidemiology Research Unit (AGE), School of Public Health, Imperial College London, London, UK; 22 Sheffield Institute for Translational Neuroscience (SITraN), University of Sheffield, Sheffield, UK; 23 German Center for Neurodegenerative Diseases (DZNE), Gehlsheimer Str. 20, 18147 Rostock, Germany; 24 Department of Psychosomatic Medicine, Rostock University Medical Center, Gehlsheimer Str. 20, 18147 Rostock, Germany; 25 Section for Dementia Research, Hertie Institute for Clinical Brain Research and Department of Psychiatry and Psychotherapy, University of Tübingen, Tübingen, Germany; 26 Institute of Cognitive Neurology and Dementia Research (IKND), Otto-von-Guericke University, Magdeburg, Germany; 27 Department of Psychiatry and Psychotherapy, Otto-von-Guericke University, Magdeburg, Germany; 28 MR-Research in Neurosciences, Department of Cognitive Neurology, Georg-August-University, Goettingen, Germany; 29 Bernstein Center for Computational Neurosciences, Charité – Universitätsmedizin, Berlin, Germany; 30 Department for Biomedical Magnetic Resonance, University of Tübingen, 72076 Tübingen, Germany; 31 Department of Psychiatry, University of Cologne, Medical Faculty, Kerpener Strasse 62, 50924 Cologne, Germany; 32 Department of Neurology, University of Bonn, Venusberg-Campus 1, 53127 Bonn, Germany; 33 Division of Neurogenetics and Molecular Psychiatry, Department of Psychiatry and Psychotherapy, Faculty of Medicine and University Hospital Cologne, University of Cologne, Cologne, Germany; 34 Department of Psychiatry & Glenn Biggs Institute for Alzheimer’s and Neurodegenerative Diseases, San Antonio, TX USA; 35 Division Translational Genomics of Neurodegenerative Diseases, Center for Neurology and Hertie Institute for Clinical Brain Research, University of Tübingen, Otfried-Müller-Strasse 27, 72076 Tübingen, Germany; 36 Luxembourg Centre for Systems Biomedicine (LCSB), University of Luxembourg, 7 avenue des Hauts Fourneaux, 4362 Esch-sur- Alzette, Luxembourg

**Keywords:** Alzheimer’s disease, Inflammation, Biomarker, Blood-based, Structural MRI, Cognition

## Abstract

**Background:**

Neuroinflammation constitutes a pathological hallmark of Alzheimer’s disease (AD). Still, it remains unresolved if peripheral inflammatory markers can be utilized for research purposes similar to blood-based beta-amyloid and neurodegeneration measures. We investigated experimental inflammation markers in serum and analyzed interrelations towards AD pathology features in a cohort with a focus on at-risk stages of AD.

**Methods:**

Data of 74 healthy controls (HC), 99 subjective cognitive decline (SCD), 75 mild cognitive impairment (MCI), 23 AD relatives, and 38 AD subjects were obtained from the DELCODE cohort. A panel of 20 serum biomarkers was determined using immunoassays. Analyses were adjusted for age, sex, *APOE* status, and body mass index and included correlations between serum and CSF marker levels and AD biomarker levels. Group-wise comparisons were based on screening diagnosis and routine AD biomarker-based schematics. Structural imaging data were combined into composite scores representing Braak stage regions and related to serum biomarker levels. The Preclinical Alzheimer’s Cognitive Composite (PACC5) score was used to test for associations between the biomarkers and cognitive performance.

**Results:**

Each experimental marker displayed an individual profile of interrelations to AD biomarkers, imaging, or cognition features. Serum-soluble AXL (sAXL), IL-6, and YKL-40 showed the most striking associations. Soluble AXL was significantly elevated in AD subjects with pathological CSF beta-amyloid/tau profile and negatively related to structural imaging and cognitive function. Serum IL-6 was negatively correlated to structural measures of Braak regions, without associations to corresponding IL-6 CSF levels or other AD features. Serum YKL-40 correlated most consistently to CSF AD biomarker profiles and showed the strongest negative relations to structure, but none to cognitive outcomes.

**Conclusions:**

Serum sAXL, IL-6, and YKL-40 relate to different AD features, including the degree of neuropathology and cognitive functioning. This may suggest that peripheral blood signatures correspond to specific stages of the disease. As serum markers did not reflect the corresponding CSF protein levels, our data highlight the need to interpret serum inflammatory markers depending on the respective protein’s specific biology and cellular origin. These marker-specific differences will have to be considered to further define and interpret blood-based inflammatory profiles for AD research.

**Supplementary Information:**

The online version contains supplementary material available at 10.1186/s13195-022-01118-0.

## Background

Biomarkers of Alzheimer’s disease (AD), such as beta-amyloid (Aβ42/Aβ40), phospho-tau-181 (p-tau-181), or neurofilament light chain (Nf-L), are found in cerebrospinal fluid (CSF) and routinely support clinical diagnostics. Research advances in recent years have seen advances in blood-based, less invasive biomarkers of Alzheimer’s disease (AD) and other neurodegenerative disorders [1]. For neuroinflammation, another AD hallmark, as well as for systemic inflammation—a potential risk factor for AD—there is still a lack of blood-based biomarkers with similar potential [2]. A large range of studies has investigated peripheral levels of inflammatory cytokines, chemokines, or complement factors in AD. Although meta-analyses attest a moderate elevation of pro-inflammatory factors in AD over the average of studies, there is tremendous variance in reported effects, including no observed differences or even higher levels in healthy controls [3, 4].

In this study, we investigated inflammatory protein serum levels and their relationship to brain structure and cognition in baseline samples of the DZNE (German Center for Neurodegenerative Diseases)—Longitudinal Cognitive Impairment and Dementia Study (DELCODE). DELCODE is a longitudinal multimodal study that focuses on individuals at risk for developing AD [5]. The panel of biomarkers consisted of immune-related markers including IL-6; IL-18; CCL2; CXCL10; MIF; YKL-40; sTREM2; sAXL; sTyro3; CRP and complement factors C1q, C3, C3b, C4, B, and H; ferritin and total ApoE protein; and two non-tau markers of neurodegeneration (FABP-3 and neurogranin). This panel was based on previous findings of our group on CSF-level effects of the respective markers [6–9]. In this previous work, these markers were characterized by their robust detectability in quantitative assays, by reproducible strong correlation to CSF tau isoform levels, between neurodegeneration and inflammation markers of the panel, and—in part—also to structural MRI and cognitive outcome data. However, data on the potential of this selection of markers as blood-based readouts is still scarce or inconclusive. Furthermore, it is unknown if effects observed for these markers in CSF are also reflected in peripheral blood and how CSF and blood levels of the markers relate to each other. Some markers of this panel, such as MIF, sAXL, and most of all complement factors, have been described to relate to AD features like amyloid burden, *APOE* status, cognition, or whole brain volume with varying results [10–28]. Subjects with high serum sTREM2 levels were reported to have an increased risk of all-cause dementia, whereas others described a significant increase of TREM2 expression in AD monocytes, but not plasma [29, 30]. A recent meta-analysis likewise did not find plasma sTREM2 levels to be altered in AD [31]. YKL-40 serum or plasma levels have been reported to correlate to CSF levels and to be increased not only in amnestic MCI, AD, and non-AD dementia but also in various other conditions, such as cancer, inflammatory diseases, infections, or coronary heart disease [32–34]. A recent study by Vergallo et al. investigated plasma YKL-40 levels in detail and found higher levels in men, a positive correlation to age, but no relation to *APOE* status [35]. Furthermore, they found a negative correlation to brain amyloid load measured by PET imaging, but no relationship with longitudinal change of amyloid load or MRI volume of hippocampal, entorhinal cortex, or basal forebrain. This body of research indicates that even for the most established AD neuroinflammation markers—such as YKL-40 or sTREM2—data on peripheral levels requires further extension to enable more definite conclusions.

Therefore, we aimed to further study the potential of the protein panel—selected based on CSF observations—as blood-based inflammatory biomarkers of AD with a focus on subjective cognitive decline (SCD) subjects as an at-risk population and the biomarker relations to CSF markers, to Braak-region-specific structural MRI measures, and cognitive performance.

## Methods

### Study design

The general design, study population, inclusion/exclusion criteria, and biomaterial sampling procedures of the DZNE DELCODE study have been described elsewhere [5]. Data on age, sex, *APOE* genotype, and body mass index (BMI), as well as on the CSF routine biomarkers (i.e., ratio Aβ42/40, phospho(p)-tau-181, total(t)-tau), T1-weighted MRI data, and neuropsychological test results, were provided by the DELCODE study management. The present study contains baseline data of 309 subjects from DELCODE including CSF-level data of the experimental biomarker panel. The sample was selected based on the availability of the experimental biomarker CSF data and the availability of serum samples for all included subjects. No other additional inclusion/exclusion criteria were applied beyond the general criteria of DELCODE [5]. This study focused on 99 subjective cognitive decline (SCD) subjects based on initial power estimates, as this group is enriched for subjects at risk or in very early stages of disease. Furthermore, 74 healthy controls (HC), 75 subjects diagnosed with amnestic mild cognitive impairment (MCI), 38 dementia of the Alzheimer’s type (DAT) subjects, and 23 cognitively normal first-degree relatives of DAT patients were included (detailed in Table 1). In brief, SCD was defined by subjectively reported cognitive decline and a test performance better than −1.5 standard deviations (SD) below normal in the CERAD neuropsychological battery. MCI was restricted to amnestic MCI with performance below −1.5 SD on the delayed recall trial of the CERAD word-list episodic memory tests. The AD group consisted of patients with mild AD and >18 points in the MMSE examination score. SCD, MCI, and AD subjects were recruited from memory clinic settings in line following clinical routine procedures. HC and relatives’ groups were recruited based on advertisements. SCD, HC, and relatives showed normal cognitive test performance (performance in all CERAD tests >−1.5SD in age, gender, and education adjusted norms) and underwent further assessments for other (non-cognitive) inclusion and exclusion criteria of DELCODE. Relatives of AD subjects are at higher risk to develop AD, show increased rates of reporting SCD compared to the average (healthy) population, and were also found to display more pronounced cognitive decline than the HC group in DELCODE [36]. Details of group definitions are provided in the DELCODE study design and baseline description [5].

**Table 1:** Cohort description

Feature	HC	Relatives	SCD	MCI	DAT	***p***
	*n* = 74	*n* = 23	*n* = 99	*n* = 75	*n* = 38	
Age (yrs.), median (SD)	68 (5)	64 (4)	71 (6)	73 (5)	75 (7)	2 × 10E^−10^
Sex (male), *n* (%)	40 (54)	17 (74)	44 (44)	23 (31)	25 (63)	4 × 10E^−4^
*APOE* (ε4 positive), *n* (%)	16 (22)	8 (35)	35 (35)	29 (39)	25 (66)	3 × 10E^−4^
BMI, median (SD)	25 (3)	26 (4)	25 (3)	26 (4)	24 (4)	0.084
Aβ42/40, median (SD)	0.097 (0.021)	0.100 (0.024)	0.095 (0.027)	0.066 (0.030)	0.046 (0.017)	9 × 10E^−15^
Aβ42/40 < 0.08, *n* (%)	17 (23)	4 (17)	36 (36)	43 (57)	35 (92)	7 × 10E^−13^
t-tau (pg/ml), median (SD)	368 (159)	372 (104)	368 (192)	483 (262)	804 (342)	7 × 10E^−13^
t-tau > 510.9, *n* (%)	15 (20)	3 (13)	21 (21)	34 (45)	31 (82)	2 × 10E^−12^
p-tau-181 (pg/ml), median (SD)	49.4 (18.4)	43.4 (12.1)	47.3 (24.4)	63.2 (31.7)	87.5 (39.6)	5 × 10E^−10^
p-tau-181 > 73.65, *n* (%)	8 (8)	0 (0)	12 (12)	24 (32)	28 (74)	6 × 10E^−17^
A− T−, *n* (%)	55 (74.3)	19 (82.6)	60 (60.6)	30 (40.5)	3 (7.9)	3 × 10E^−17^
A− T+, *n* (%)	2 (2.7)	0 (0.0)	3 (3.0)	2 (2.7)	0 (0.0)	
A+ T−, *n* (%)	13 (17.6)	4 (17.4)	27 (27.3)	20 (27.0)	7 (18.4)	
A+ T+, *n* (%)	4 (5.4)	0 (0.0)	9 (9.1)	22 (29.7)	28 (73.7)	
PACC5 score, median (SD)	0.57 (0.41)	0.31 (0.65)	0.38 (0.50)	−0.74 (0.64)	−1.93 (0.57)	< 1 × 10E^−15^
Mean follow-up time (yrs., SD)	3.5 (1.1)	3.0 (1.4)	3.5 (1.3)	2.7 (1.4)	2.0 (1.4)	2 × 10E^−6^
Braak I, median (SD)	0.28 (0.66)	0.43 (0.46)	0.28 (0.73)	−0.05 (1.21)	−1.14 (1.05)	6 × 10E^−12^
Braak II, median (SD)	0.43 (0.72)	0.41 (0.73)	0.34 (0.74)	−0.37 (0.95)	−1.52 (0.92)	< 1 × 10E^−15^
Braak III, median (SD)	0.32 (0.55)	0.25 (0.47)	0.28 (0.50)	−0.32 (0.67)	−0.55 (0.94)	5 × 10E^−14^
Braak IV, median (SD)	0.29 (0.51)	0.25 (0.44)	0.07 (0.48)	−0.28 (0.66)	−0.58 (0.80)	6 × 10E^−12^
Braak V, median (SD)	0.30 (0.48)	0.43 (0.40)	0.13 (0.53)	−0.24 (0.65)	−0.55 (0.79)	3 × 10E^−11^
Braak VI, median (SD)	0.11 (0.72)	0.25 (0.67)	0.05 (0.65)	−0.34 (0.80)	−0.04 (0.98)	0.001

The table describes demographic features, AD routine biomarkers, AT classification composition, cognitive performance (PACC5) score with follow-up period, and the structural Braak ROI scores between the screening diagnosis groups of the DELCODE sub-cohort used in this study. By DELCODE screening diagnosis, the cohort included 74 cognitively healthy controls (HC), 99 subjective cognitive decline (SCD) subjects, 75 mild cognitive impairment (MCI) subjects, 38 patients with dementia of the Alzheimer’s type (DAT) subjects, and 23 cognitively normal first-degree relatives of DAT patients. Diagnosis groups with more pronounced clinical phenotype had significantly larger fractions of AT biomarker-positive subjects. Continuous features (e.g., age) were compared by the Kruskal–Wallis test and categorical variables (e.g., sex) by the chi-square test

Biomarker panel proteins were determined in serum and analyzed together with the available data to describe interrelations to their CSF counterparts and routine AD CSF markers. Furthermore, serum protein levels were tested for their relation to subject groups defined by screening diagnosis or AD routine CSF biomarker patterns, structural imaging scores, and cognitive outcome. All analyses were adjusted for age, sex, *APOE* status, and body mass index (BMI) as potential confounders of systemic immune responses. Bivariate relations between these covariates and the serum biomarker levels are provided in Additional file 1: Table S1. Statistical models were run with different adjustment variants as well as with exclusion of MCI and AD subjects from the whole cohort, as detailed below.

### Biomarker acquisition

Serum levels of the experimental biomarker panel (IL-6, Il-18, CCL2, CXCL10, MIF, YKL-40, sTREM2, sAXL, sTyro3, CRP and complement factors C1q, C3, C3b, C4, B, and H, ferritin and total ApoE protein, as well as the non-tau markers of neurodegeneration FABP-3 and neurogranin) were determined utilizing a series of immunoassays on different platforms, optimized for the individual marker’s dilution range and multiplexing capacity of the respective immunoassay technique (details provided in Table 2). To avoid multiple freeze-thaw cycles during measurements, samples were thawed initially on ice, split into smaller aliquots, and stored in 96 well V-bottom storage plates (Greiner Bio-One, ref. 651101), sealed with a freezing-resistant aluminum foil (Greiner Bio-One, ref. 676090), at −80 °C until analysis. This way, the total number of freeze-thaw cycles of samples was 2 at the time of immunoassay conduction. The maximum accepted coefficient of variance (CV) for samples run in duplicate was 20%. Samples with higher CV underwent repeated measurement. An internal, aliquoted reference serum sample was used to control for inter-run variances.

**Table 2:** Immunoassay specifications

Protein	Assay CV (median, SD, min–max)	Single-/multiplex	Vendor	Assay method	Product no.	Dilution (x-fold)
**FABP-3**	3.4 ± 2.9 0.0–15.1	3-plex	Merck KGaA	Bead-based (Luminex Corp.)	HNS2MAG-95K	5
**Neurogranin**	2.6 ± 3.0 0.0–17.2					
**Ferritin**	3.7 ± 4.5 0.0–19.8					
**C1q**	2.2 ± 2.8 0.0–18.6	6-plex			HCMP2MAG-19K	4000
**C3**	1.6 ± 2.4 0.0–15.8					
**C4**	1.6 ± 2.3 0.0–19.6					
**Factor B**	2.0 ± 2.9 0.0–19.2					
**Factor H**	1.7 ± 2.5 0.0–18.8					
**C3b**	3.7 ± 4.3 0.0–19.8					
**sAXL**	1.3 ± 1.8 0.0–16.8	Singleplex	R&D Systems, Inc.	Colorimetric	DY154	250
**sTyro3**	2.0 ± 2.5 0.0–16.5	Singleplex			DY850	6
**YKL-40**	1.2 ± 1.8 0.0–19.4	Singleplex			DY2599	200
**IL-6**	1.9 ± 1.7 0.0–10.0	Singleplex	Quanterix Corp.	Digital ELISA (SIMOA)	101622	5
**Il-18**	2.1 ± 2.0 0.0–10.0	Singleplex			102700	500
**CXCL10**	2.2 ± 2.2 0.0–11.3	3-plex	Meso Scale Diagnostics, LLC.	Electrochemi-luminescence	U-PLEX K15047K and K15046K	15
**CCL2**	1.7 ± 1.5 0.0–10.4					
**MIF**	1.2 ± 1.6 0.0–18.2					
**CRP**	1.8 ± 1.7 0.0–11.4	Singleplex			K15198D	1000
**ApoE (total)**	3.5 ± 3.7 0.0–17.3	Singleplex			F212I	4000
**sTREM2**	4.2 ± 4.3 0.0–18.8	Singleplex			Homebrew	5

The table displays details on the single- and multiplex ELISA kits used. Methods for the homebrew sTREM2 ELISA have been described elsewhere [37]. Assay CV reports the median coefficient of variance with standard deviation, minimum, and maximum. The maximum accepted CV was 20%; samples exceeding this limit underwent repeated measurement (see the “Methods” section)

### Correlation and group-wise analyses

IBM SPSS Statistics 21 (IBM Corporation, Armonk, USA) was used to check the influence of potential confounders on the experimental inflammatory biomarkers (Additional file 1: Table S1) and to calculate partial Spearman correlation matrices as well as covariate-controlled group-wise comparisons (further details on statistics and results provided in Additional file 1: Tables S2-S4). Correlation matrices were calculated in three models: (1) the whole cohort with adjustment for covariates age, sex, *APOE* status, and BMI; (2) additional exclusion of MCI and AD subjects to verify if effects persist in subjects without objective cognitive impairment; (3) same as 1, with additional adjustment for CSF Aβ42/Aβ40 and CSF p-tau-181, to test if effects in the whole cohort persisted beyond adjustment for routine AD pathology marker levels. Cut-off values for routine AD biomarker-based subjects groups were based on Gaussian mixture modeling of the DELCODE data independent of any group assignments using the R package flexmix (version 2.3-15) [38]: amyloid ratio (A) Aβ42/Aβ40 0.08, tau pathology (T) by p-tau-181 73.65 pg/ml, and neurodegeneration (N) by t-tau 510.9 pg/ml. As both tau isoform CSF levels are highly correlated in DELCODE, we did not apply the A/T/N scheme exactly but used a similar approach of A/T and A/N as complementary measures of pathological amyloid/tau isoform biomarker combination profiles. Group-wise comparisons were adjusted for multiple testing using the Bonferroni method. For significant group-wise comparisons, receiver operating characteristics (ROC) was calculated in Prism 8 (GraphPad Software Inc., La Jolla, USA) to determine the area under the curve (AUC) with a 95% confidence interval.

### Structural measure analysis

Thickness and volume measurements were obtained by segmentation of the T1-weighted MR images with Freesurfer 6.0 (Fischer et al., http://surfer.nmr.mgh.harvard.edu/) using the Desikan–Killiany atlas [39, 40]. MRI data were available for 267 subjects (87% of the cohort). Six structural measures were derived by combining individual regions into a priori composite regions of interest (ROI) [41, 42] that mirror Braak stages of neurofibrillary tangle pathology [43]. Earlier Braak regions show earlier atrophy in the course of AD and thus should most strongly correlate with AD pathology. The six Braak ROIs covered the following regions: I: entorhinal cortex; II: hippocampus; III: amygdala, fusiform, parahippocampal cortex, lingual; IV: temporal regions, cingulate, retrosplenial cortex, insula; V: frontal and parietal regions; VI: primary visual, motor, and sensory areas; exact list of individual regions described by Baker et al. [42]. Volume measures were used for subcortical ROIs (hippocampus, amygdala), which were adjusted for intracranial volume, and thickness measures were used for all cortical regions. For each Braak composite region, individual thickness/volume measures were *z*-scored across the whole sample and then averaged. We calculated bilateral means as we had no hemisphere-specific hypotheses.

To assess the relations of the AD-related inflammatory markers to Braak ROI structural measures, bivariate Spearman rank correlations were calculated using R between each of the markers and Braak structural measures. For comparison of effect strength, this analysis was also performed for the CSF amyloid and tau biomarkers. Structural measure correlations were run in three different adjustment models: (1) the whole cohort with adjustment for covariates age, sex, *APOE* status, and BMI; (2) additional exclusion of MCI and AD subjects to verify if effects persist in subjects without objective cognitive impairment; (3) same as 1, with additional adjustment for CSF Aβ42/Aβ40 and p-tau-181, to test if effects in the whole cohort persist beyond adjustment for routine AD pathology marker levels.

### Analysis of cognitive decline in the PACC5 score

The Preclinical Alzheimer’s Cognitive Composite (PACC5) [44] was used in this study to assess change in cognitive function over time. It was developed to sensitively track cognitive decline in the early phase of AD. Here, it was derived by *z*-standardizing (based on means and standard deviations from all cognitively normal individuals in DELCODE) and averaging the free cued and selective reminding test (FCSRT) total and free recall, symbol digit modalities test (SDMT), logical memory delayed recall, semantic fluency (sum of animals and grocery named in 1 min), and the Mini-Mental State Examination (MMSE).

We used linear mixed models with a latent process as implemented in the R package lcmm [45, 46] to model the baseline levels and change in the PACC5 over up to 5 years in DELCODE. Herein, beta cumulative distribution function was used as it accounts for the curvilinearity and non-equal interval scaling that is frequently observed for neuropsychological outcomes [45]. A univariate latent process linear mixed model with a random intercept and random slope of time from baseline was modeled. We examined the fixed effect of markers on the PACC5 at baseline (main effect) and the interaction of time from baseline to study effects on change over time. The mean follow-up time across all participants included in the analysis was 3.1 years (SD = 1.4). Of 289 subjects with PACC5 score data, 34 (11.8%) had provided only a baseline assessment. We included all subjects in the longitudinal analysis, irrespective of whether or not they provided a follow-up assessment beyond the baseline in order to maximize power to detect differences in cognition at baseline and avoid selection bias due to the exclusion of participants. Missing observations were handled by full maximum likelihood estimation in the mixed models. Markers were *z*-standardized (based on sample mean and standard deviation) to ease comparisons across markers. Again, the three different adjustment models were used: (1) the whole cohort with adjustment for covariates age, sex, *APOE* status, and BMI (fixed effects for main effects and interaction with time); (2) additional exclusion of MCI and AD subjects to verify if effects persist in subjects without objective cognitive impairment; (3) same as 1, with additional adjustment for CSF Aβ42/Aβ40 and p-tau-181 (fixed effects for main effects and interaction with time), to test if effects in the whole cohort persist beyond adjustment for proxies of AD pathology.

### Biomarker transcriptome and proteome data

Data of the Human Protein Atlas (https://www.proteinatlas.org/) was retrieved to support literature-based discussion of protein origins in serum. Data extracted from the atlas included brain, blood, and tissue RNA as well as tissue protein levels (see Additional file 1 for details) [47–51].

## Results

### Interrelation between serum and CSF biomarkers

**Fig. 1:**
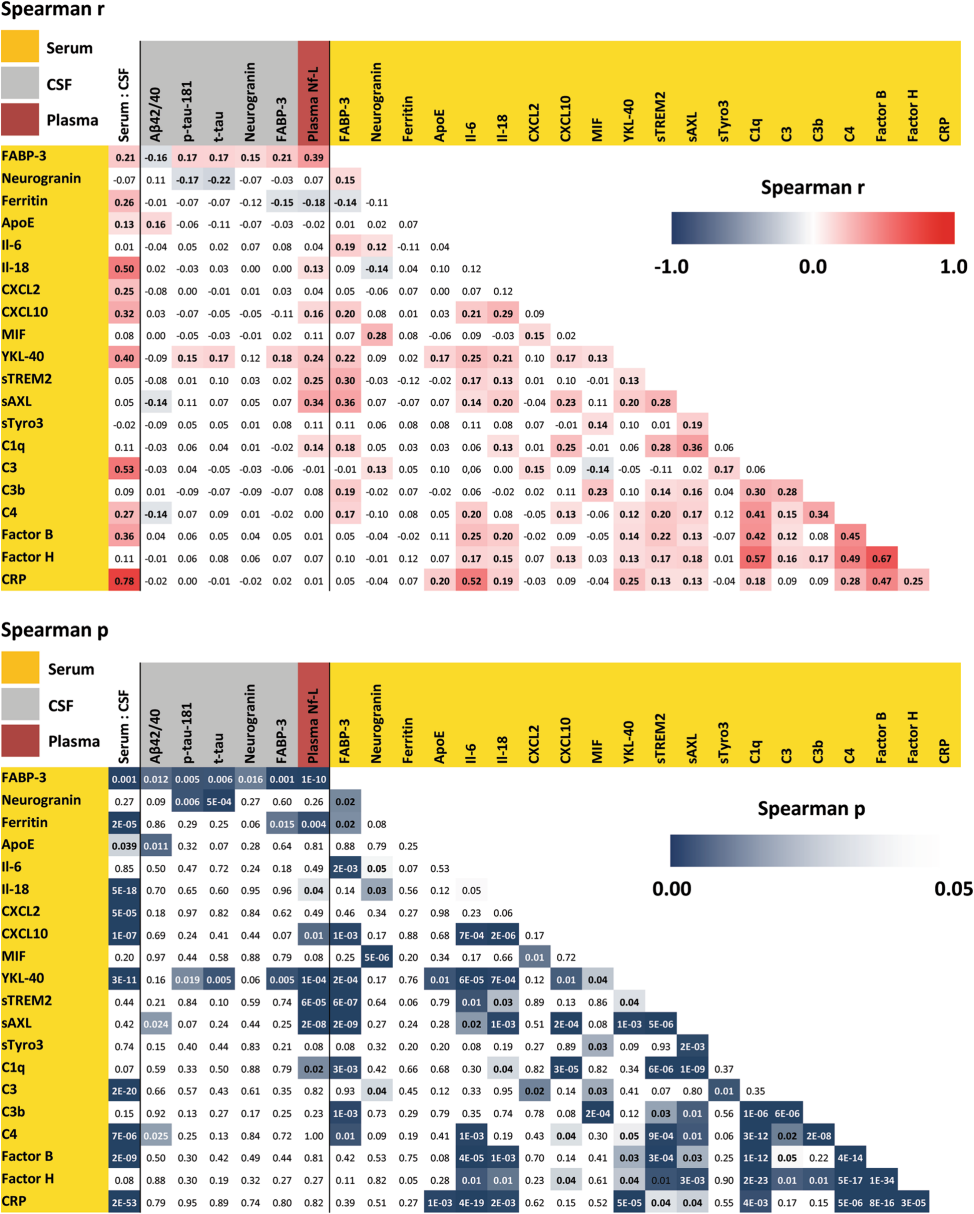
Serum biomarker correlation matrix. Partial Spearman correlation matrix adjusted for covariates age, sex, BMI, and *APOE* status. Serum biomarker levels (yellow) were correlated against their CSF counterparts (CSF: serum), to CSF AD biomarkers (gray, Aβ42/40 ratio, p-tau-181, t-tau, neurogranin, and FABP-3), as well as to plasma levels of Nf-L determined in DELCODE (red). The upper panel depicts the strength of correlation (Spearman *r*), and the lower panel, significance (Spearman *p*). Between CSF and serum, several proteins showed weak to modest correlations, whereas others were not correlated at all. The strongest correlation was observed between serum and CSF CRP protein. FABP-3, ApoE, sAXL, and complement C4 showed weak correlations to amyloid. FABP-3 and YKL-40 were consistently correlated to CSF levels of tau isoforms and neurodegeneration markers. Plasma levels of Nf-L were correlated to several proteins from the serum panel, even if the respective proteins did not relate to their CSF counterparts or to CSF AD markers

We first aimed to assess how serum biomarkers would relate to their corresponding CSF levels and routine AD biomarkers (Fig. 1). This analysis also included interrelations between the investigated serum markers. The biomarker panel contained two non-tau neurodegeneration markers (FABP-3 and neurogranin) which yielded differing associations: serum FABP-3 showed weak, but consistent positive correlations to CSF FABP-3 as well as to CSF levels of tau isoforms and neurogranin, and was also negatively correlated to CSF Aβ42/40 ratio (Spearman r (value and 95% CI) ranging from 0.15; CI 0.04–0.26 to 0.21; CI 0.10–0.31). In contrast, there was no correlation for neurogranin between serum and CSF; however, serum neurogranin was inversely related to CSF tau isoform levels (*r* = −0.17; CI −0.28 to −0.06 and *r* = −0.22; CI −0.32 to −0.11), but not to Aβ42/40 or CSF FABP-3.

Regarding the inflammatory markers, a particular high correlation between corresponding serum and CSF levels was observed for CRP (*r* = 0.78; CI 0.73–0.82). Nine other inflammation-related markers (ferritin, ApoE, Il-18, CCL2, CXCL10, YKL-40, complement C3, C4, and factor B) showed positive correlations of differing strength (lowest *r* = 0.13; CI 0.02–0.24 for ApoE, highest *r* = 0.53; CI 0.45–0.61 for complement C3), whereas the eight other inflammatory markers did not correlate significantly between serum and CSF.

Regarding the relationships between CSF-based routine AD markers and inflammatory serum proteins, three serum proteins (ApoE, sAXL, and C4) showed a relation to CSF Aβ42/40 ratio. For only one marker—YKL-40—positive relations to tau isoforms and CSF FABP-3 were found. Serum ferritin was inversely related to CSF FABP-3, but not to CSF tau. These relations between pro-inflammatory serum and CSF AD markers were significant, but showed low effect sizes (*r* = 0.14; CI 0.03–0.25 to 0.18; CI 0.07–0.29).

DELCODE also provides measures of plasma Nf-L, one of the best-established peripherally accessible neurodegeneration markers of axonal degeneration, with higher NfL levels reflecting a higher rate of axonal decay. Plasma Nf-L levels were most of all positively related to serum levels of several experimental markers (FABP-3, Il-18, CXCL10, YKL-40, sTREM2, sAXL, C1q), with the strongest for FABP-3 (*r* = 0.39; CI 0.29–0.48) and the least strong observed for Il-18 (*r* = 0.13; CI 0.02–0.24).

In terms of interrelations of serum panel markers to each other, we observed several mostly positive correlations between serum FABP-3 and inflammatory markers (*r* = 0.15; CI 0.04–0.26 to *r* = 0.36; CI 0.26–0.45). We also found a large number of weak to moderate strength positive interrelations between markers of the immune-related and complement part of the panel, of which the strongest were between IL-6 and CRP (*r* = 0.52; CI 0.43–0.60) and between complement factor H and C1q (*r* = 0.57; CI 0.49–0.64) as well as factor B (*r* = 0.67; CI 0.60–0.73).

Next, we tested two alternative correlation models to examine the influence of AD stage and pathology on the relations between serum and CSF biomarkers. In the first model, we examined effects in pre-dementia subjects by excluding MCI and AD subjects from the cohort but retaining the HC, SCD, and relatives’ subjects. In the resulting model, there was little effect on each marker’s corresponding serum to CSF correlations (Additional file 1: Fig. S1A). However, the relations of serum FABP-3 and YKL-40 to CSF Aβ42/40, tau isoforms, neurogranin, and FABP-3 were not significant in this model, whereas the correlation of serum ApoE to CSF Aβ42/40 remained significant. Furthermore, most of the previously significant correlations between experimental serum markers (FABP-3, Il-18, CXCL10, YKL-40, sTREM2, sAXL, but not C1q) and plasma-NfL remained nearly unaffected by the exclusion of MCI and AD subjects.

As the exclusion of subjects reduces the power of analysis, we also calculated a second alternative model, including the full cohort but adjusting for CSF Aβ42/40 and p-tau-181 as proxies of AD pathology. In this model, the serum to CSF relations of the individual markers remained again largely unchanged (Additional file 1: Fig. S1B). The previously observed relations between serum FABP-3 and CSF neurogranin were nonsignificant in this model, and the relation between serum YKL-40 and CSF FABP-3 also lost significance (*p* = 0.07). Again, experimental inflammatory serum marker to plasma Nf-L correlations remained robust in this model.

In summary, several inflammatory serum markers correlated to varying extent to their CSF counterparts. These relations did not change when excluding objectively impaired subjects or adjusting for CSF AD biomarker levels. Likewise, observed relations between inflammatory serum markers and plasma Nf-L were robust in the different models. Serum ApoE was the serum marker most consistently linked to CSF Aβ42/40 levels. Serum YKL-40 showed the most consistent correlations to CSF tau isoforms and other neurodegeneration biomarker levels. However, these were not robust against the exclusion of objectively impaired cases or adjustment against AD CSF routine marker levels.

### Group-wise comparisons

**Fig. 2:**
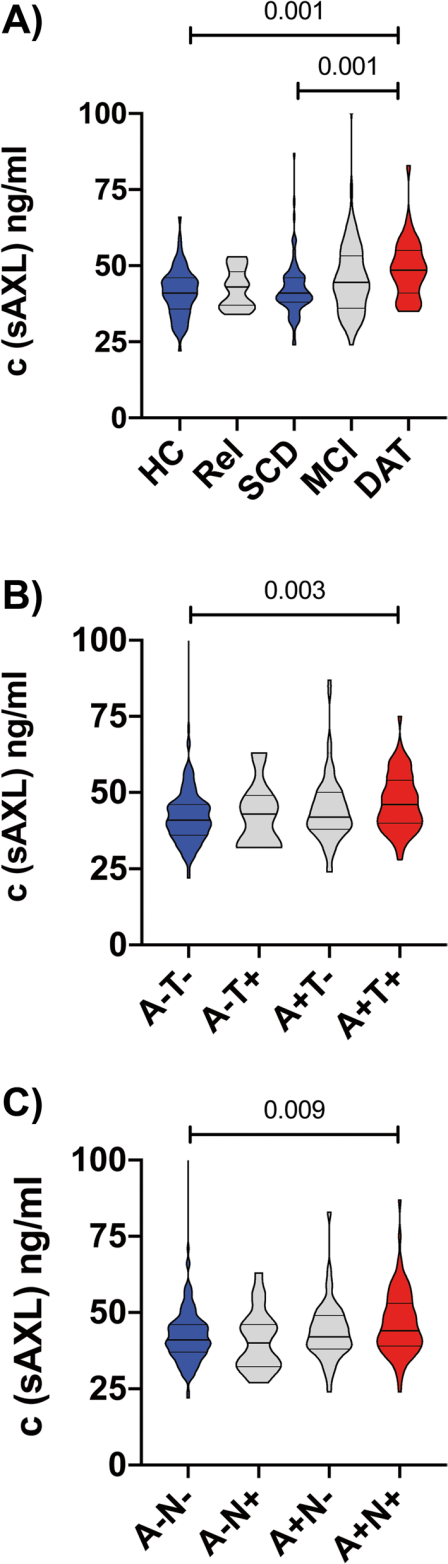
Elevation of serum sAXL in biomarker-positive DAT subjects. Differences of sAXL serum levels between subject groups based on screening diagnosis or CSF biomarker profile. Violin plots with median and interquartile range. Groups colored red were elevated against groups colored blue. Gray color indicates indifferent groups. Further statistical details are described in Additional file 1: Tables S2-S4. Serum sAXL levels were elevated in DAT versus SCD or HC subjects (**A**), as well as in either p-tau-based A+T+ subjects (**B**) or t-tau-based A+N+ subjects (**C**) against A−T− or A−N− subjects, respectively. There was no significant elevation in either MCI subjects or those with only amyloid or tau isoform-positive CSF biomarker profile

We next assessed whether serum biomarkers differed between screening diagnoses and whether they relate to AD biomarker-based schematics (see Additional file 1: Tables S2 to S5 for details on respective statistics). For this purpose, we used Aβ42/40 ratio as a CSF marker for cerebral amyloid (A) deposition, combined with either p-tau-181 as a marker of tau pathology (T) or total tau as a marker of neurodegeneration (N) [52]. As DELCODE is an AD-focused cohort, A/T and A/N groups generated by this approach were largely redundant, limiting the application of the full A/T/N schematic but nonetheless enabling comparison of results between the two tau isoforms. Only one marker of the panel, serum sAXL, differed significantly between screening diagnoses, as well as in the biomarker-based schematics (Fig. 2). Specifically, the elevation of sAXL was strongest in DAT subjects compared to SCD and HC, and otherwise only elevated in subjects with full AD profile (A+T+ or A+N+, respectively). If combining both screening diagnosis and the biomarker-based classification into a biomarker-stratified clinical setting (HC A−T−, versus “AD spectrum”: SCD, MCI, and AD combined if A+ T− or A+T+)—therefore excluding any biomarker-positive HC as well as suspected non-AD subjects from the comparison—we also observed elevation of sAXL in the stratified AD spectrum versus control subjects (Additional file 1: Table S5). This is in line with the increasing fraction of A/T biomarker-positive subjects in DAT compared to HC and SCD groups (Table 1). Receiver operating characteristic (ROC) calculation was performed for all respective group-wise comparisons and provided the following area under the curve (AUC) results (with 95% CI) for sAXL: AD vs. HC 0.73 (0.63 to 0.83); AD vs. SCD 0.72 (0.62 to 0.82); A+T+ vs. A−T− 0.65 (0.60 to 0.73); A+N+ vs. A−N− 0.63 (0.55 to 0.70); and stratified AD spectrum vs HC 0.73 (0.64 to 0.81). Less robust changes—not passing multiple testing correction—were observed for CRP in MCI against SCD, for FABP-3 in AD vs. SCD, for neurogranin in A+N− against A+N+, and for C3b and MIF in the stratified AD spectrum setting (Additional file 1: Tables S2 to S5).

### Relationship of serum inflammatory markers with structural MRI

**Fig. 3:**
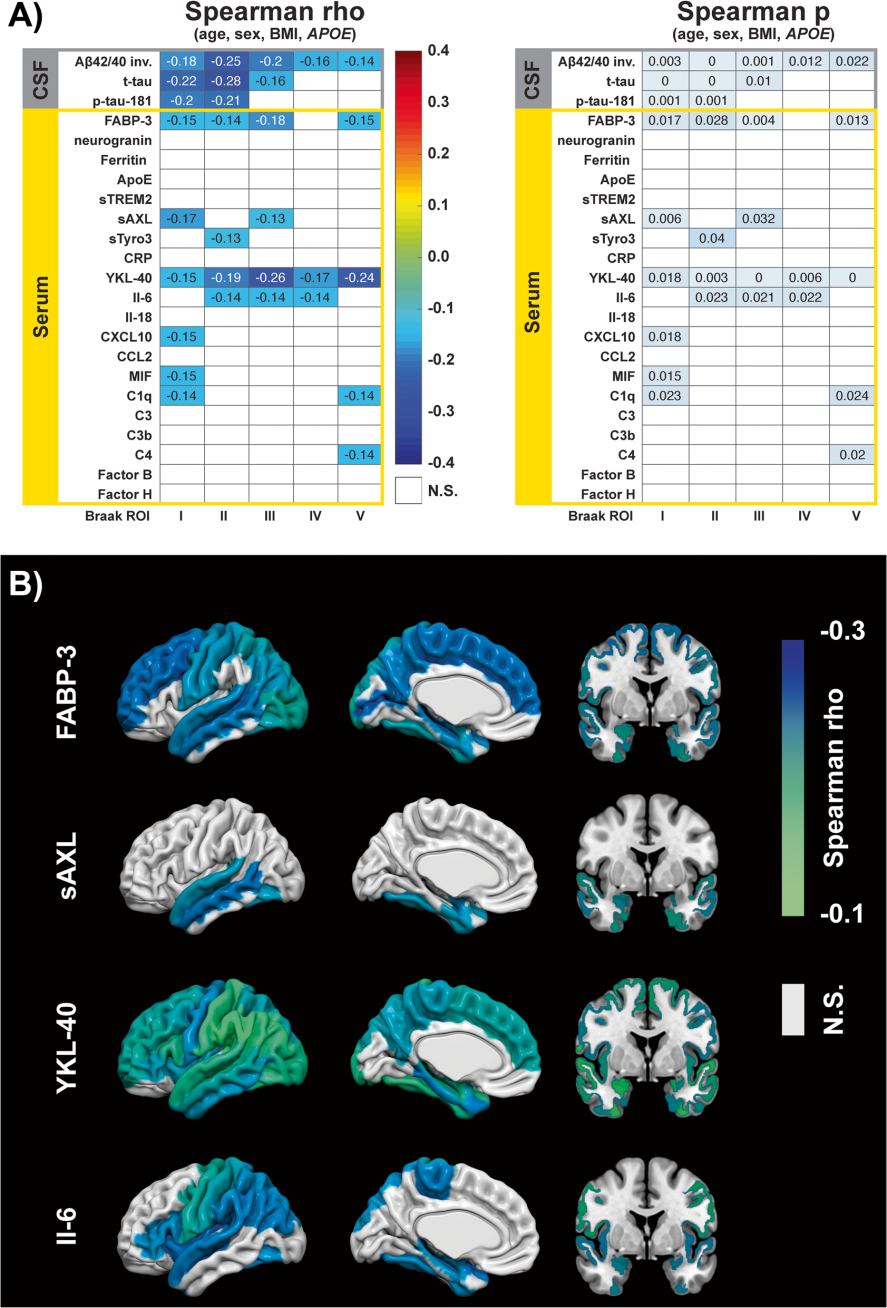
Relations of serum biomarkers to structural MRI. **A** Bivariate Spearman partial correlation matrix calculated for serum markers versus Braak stage composite scores of brain structure, adjusted for age, sex, BMI, and *APOE* status (yellow box). Correlation matrices (left panel) show the strength (rho) of all correlations that were significant at uncorrected *p* value < 0.05 (right panel). Results for CSF AD markers (Aβ42/40 ratio, tau) are depicted for comparison purposes (gray box). The ratio Aβ42/40 was inverted to visualize how more pathological amyloid levels relate to lower structural scores. All significant serum markers showed negative relations to structure, of which FABP-3, YKL-40, and IL-6 were most consistent. Aside from these, only sAXL and C1q were related to more than one Braak region. Several correlations were also observed for single markers in Braak stage I, consistent with results of a multiple regression, using serum inflammation markers and predicting structure, significant only for the Braak stage I ROI, explaining 12% of the variance in this region. *p* values of “0” in the right panel denote *p* < 0.001. **B** Freesurfer-region specific, bivariate Spearman relations of FABP-3, sAXL, YKL-40, and IL-6. Correlation strength (rho), at uncorrected *p* > 0.05 for illustration purposes, is plotted rendered on the brain surface. All correlations were adjusted for age, sex, BMI, and *APOE* status

Relations between serum inflammatory markers and structural MRI were first analyzed by multiple regression models including all inflammatory markers as predictors for brain structure in each of six Braak regions of interest (ROI) summarized in composite scores. Only for the Braak I ROI, the multiple regression model was significant (*F* (16, 240) = 2.27, *p* =.004), with 12% of variance in this region explained by serum inflammatory markers after controlling for age, sex, BMI, and *APOE* status. Consistent with this result, bivariate relations of the individual markers with structure (Fig. 3A) were most frequently observed for Braak I, including negative relations to structure for serum sAXL, YKL-40, CXCL10, MIF, and C1q. All correlations were negative with higher marker levels being related to reduced thickness in the Braak I ROI (i.e., entorhinal cortex). Four of the analytes also showed consistent negative relations to more than one Braak ROI (sAXL, YKL-40, IL-6, and C1q). Of these, YKL-40 was most striking, with significant relations in all five Braak ROI and effect strength comparable to that of CSF routine AD markers. We furthermore observed significant negative relations of serum FABP-3 to four of the Braak ROI, but none for serum neurogranin.

When excluding MCI and AD subjects from the cohort however, the observed consistencies were lost and only arbitrary relations remained, with exception of the strongest YKL-40 relations to Braak ROI III and V (Additional file 1: Fig. S2A). Adjustment of this analysis against CSF levels of the AD biomarkers Aβ42/40 and p-tau-181 had little effect on the results (Additional file 1: Fig. S2B).

The regional pattern of relations of FABP-3, sAXL, YKL-40, and IL-6 with brain structure is further visualized in Fig. 3B. Here, the correlation strength for individual Freesurfer brain regions that compose the Braak ROIs is displayed, showing that higher serum levels were related to lower structural integrity in medial temporal regions for all 3 markers and most parts of the brain for YKL-40.

### Cognitive outcome analysis

**Fig. 4:**
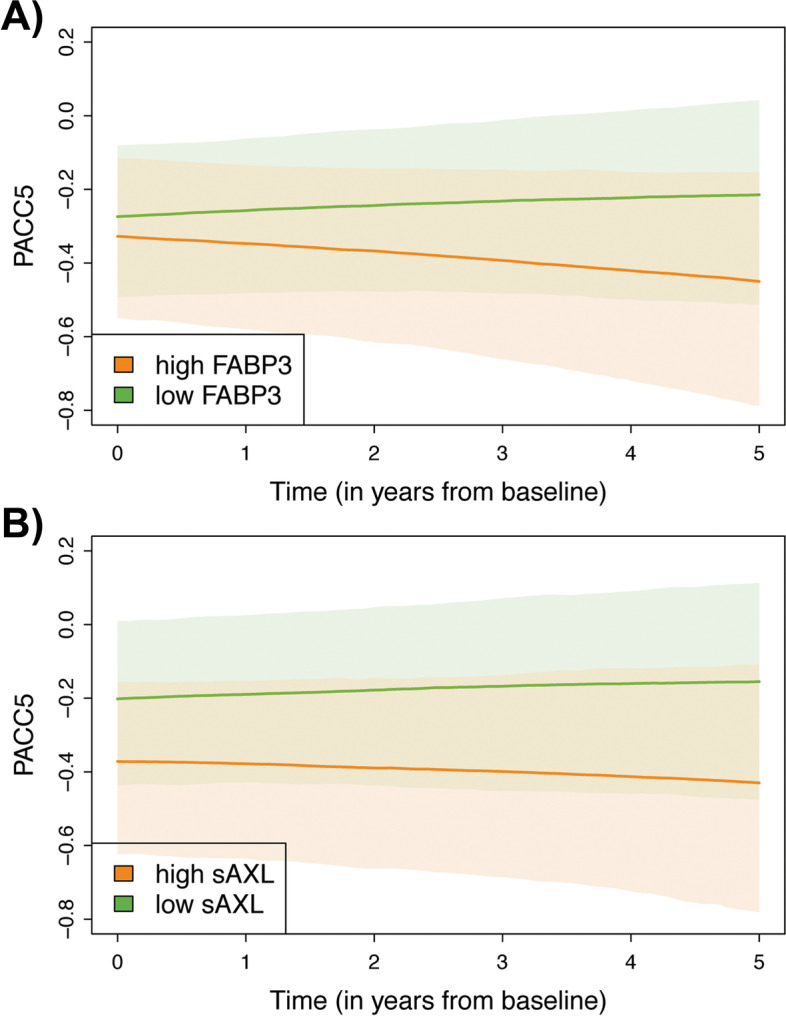
Serum biomarker relations to the longitudinal outcome. Serum biomarker levels were correlated against longitudinal PACC score values and plotted as one standard deviation (SD) above and below the mean standardized biomarker level. Available subject numbers throughout follow-ups were as follows: baseline, *N* = 289; Y1, 245; Y2, 213; Y3, 164; Y4, 89; Y5, 20. Correlations are displayed with confidence intervals. Results are adjusted for age, sex, BMI, and *APOE* status. Serum FABP-3 did not differ at baseline, but higher baseline levels predicted cognitive decline at follow-up (**A**, *p* = 0.003). In contrast, sAXL (**B**) did not predict change over time, but differed at baseline, with higher levels in subjects with stronger cognitive impairment (*p* = 0.003)

Three serum markers were significantly related to cognition as measured by PACC5: sAXL, ApoE, and FABP-3 (statistical details are provided in Additional file 1: Table S6, and significant findings are displayed in Fig. 4 for sAXL and FABP-3). Higher sAXL and ApoE were related to lower cognitive performance at baseline, but did not predict changes. In contrast, FABP-3 did not relate to cognition at baseline, but higher baseline FABP-3 was indicative of cognitive decline at follow-up.

Adjustment against CSF levels of the AD markers Aβ42/40 and p-tau-181 had no major effect on the results for sAXL, ApoE, and FABP-3 (Additional file 1: Table S7, model A). However, the effects became nonsignificant when excluding the MCI and AD subjects from the cohort (Additional file 1: Table S7, model B).

## Discussion

This study provides data on the interrelations between serum inflammation biomarkers and routine AD biomarkers, brain structure data, and cognitive outcome in a cohort with emphasis on potential early stages of AD, such as SCD. The relevance of these findings is underlined by the fact that experimental inflammatory serum markers were correlated to plasma levels of Nf-L, a marker of CNS axonal degeneration and decay. Furthermore, our analysis identified sAXL, IL-6, and YKL-40 as the three most prominent inflammatory markers. Noteworthy, most effects observed for these markers were robust against adjustments for the AD standard CSF biomarkers Aβ42/40 and p-tau-181. These results can be interpreted in the way that the respective serum markers provide information beyond CSF AD marker levels. As such, they might reflect inflammatory processes independent of beta-amyloid or tau within the cascade of AD. The same effects were less robust against the exclusion of MCI and AD subjects from the cohort, though. Therefore, it is possible that these observations are driven by later disease stages, in line with the elevation of serum sAXL observed in DAT and A+/T+ subjects. However, as the exclusion of MCI and AD subjects from the cohort also reduces power, this possibility cannot be confirmed with certainty in our study.

In many ways, the effects observed for these three proteins were comparable to those found for FABP-3. While FABP-3 itself is not functionally related to inflammation, it showed some correlation to different inflammatory markers in serum and shares some other features with the inflammation panel markers investigated in this study: First, CSF FABP-3 was reproducibly described previously as a neuronal damage biomarker in Alzheimer’s disease. Second, our data also indicate that blood-based FABP-3 may have prognostic potential as it predicted future cognitive decline beyond standard CSF AD biomarkers. However, previous studies found elevated peripheral FABP-3 levels in dementia with Lewy bodies, but not AD [53–55]. Similar to inflammatory markers, FABP-3 is not exclusively expressed in the brain (see Additional file 1: AF3–AF6 for an overview of Human Protein Atlas data). Instead, FABP-3 is found at RNA and protein levels in various organs, including T-cells, muscle tissue, and kidney, with the by far largest source described in literature being secretion from cardiac and skeletal muscle tissues as a myokine [6, 56–58]. We observed significant, but weak correlations between serum and CSF FABP-3, as well as to CSF amyloid, tau, and neurogranin. Serum FABP-3 was also inversely related to Braak ROI structural measures, and finally, higher baseline FABP-3 was related to longitudinal worsening in cognitive performance. The similarities in significance and strength of effect found for FABP-3 and inflammatory markers indicate that both have similar potential and limitations in serum, if the respective protein is not exclusively or at least highly specifically expressed in the brain. If there is a shared mechanism between changes of serum levels of FABP-3 and inflammatory markers in AD is speculative to this point: The number of correlations observed in our study between FABP-3 and various immune markers could indicate some co-regulation, but the strength of these correlations was too low to point towards specific interactions.

Of the three prominent inflammatory markers, AXL is expressed in various organs including the brain, heart, skeletal muscle, liver, kidney, male and female reproductive system, and digestive tract and has functions primarily in the clearance of apoptotic cells by phagocytes, immune regulation, platelet activation, and vascular permeability in liver endothelial cells (Additional file 1: AF3-6) [59–61]. It is established that in the brain AXL—just as the other TAM receptors, Tyro3 and Mertk—has functions in both, regulating innate immune responses as well as neuroprotection [62–65]. The soluble ectodomain of AXL is derived from proteolytic cleavage, and CSF levels of sAXL have been reported as biomarkers of AD, relating to markers of amyloid and neurodegeneration but also larger structure and, to some extent, improved cognition [7, 9, 66]. For the periphery, sAXL has been described as a blood biomarker of liver fibrosis, rheumatoid arthritis, and, most of all, various types of neoplasms [59–61]. While AXL signaling is considered to be immuno-regulative, details on its function as well as the implications of AXL shedding remain incompletely understood: Increased sAXL levels are thought to represent overexpression of AXL in tumor tissue, whereas in arthritis, lower levels of sAXL are observed. These findings suggest that sAXL levels are a proxy of cellular receptor expression. In the present study, higher serum sAXL was mildly inversely correlated to more pathological CSF amyloid ratio as well as to lower structure in Braak I and III regions, but not to CSF sAXL levels. Furthermore, sAXL was the only marker showing elevation in group-wise comparisons, in particular in DAT compared to HC and SCD groups. Though serum sAXL was not related to CSF tau isoforms or other neurodegeneration markers, the A/T and A/N schematic results indicate that the observed elevation was driven by subjects who were positive on all three A/T/N parameters, compared to those with normal amyloid or tau markers. Finally, higher sAXL levels were related to poorer cognitive function at baseline, but they were not significantly related to change over time. Overall, these results indicate that serum sAXL is mildly elevated in AD subjects with full AD pathology and already low cognitive performance. If this elevation represents spillover from the CSF in late disease stages or if it is caused by the incompletely understood peripheral regulation of AXL, secondary to CNS damage, remains to be resolved.

IL-6 is a pleiotropic cytokine expressed by macrophages, dendritic cells, lymphocytes, endothelial cells, osteoclasts, and hepatocytes [67–69]. It has a broad range of functions in physiology, metabolism, aging, development, exercise, angiogenesis, osteoclastogenesis, cell differentiation, trauma, and acute and chronic inflammation and has been reported in several disease areas including cancer, autoimmune disease, cognitive dysfunction, and psychiatric disorders. Noteworthy, in the matched CSF data of this cohort, we did not observe significant effects in clinical or AT/N biomarker group test schematics on IL-6 and there was no correlation between serum and CSF IL-6 (*p* = 0.85, Fig. 1). Furthermore, serum IL-6 did not relate to cognitive function at baseline or longitudinal change. Previous studies frequently, but not consistently reported the relation of peripheral IL-6 to cognitive performance, and IL-6 did not relate to the TSPO-PET-imaging marker of neuroinflammation [70–73]. Yet, in our study, we found a negative relation between serum IL-6 and structural MRI measures in the Braak ROI II–IV. This is in line with several previous reports that described inverse correlations of IL-6 to total cranial brain volume, though findings vary on its relation to perivascular spaces, white matter hyperintensities, and hippocampal volume [72–83]. In part, our results also match those of McCarrey et. al. who found serum IL-6 levels to correlate with cortical thinning in non-demented subjects over a period of 7.5 years, whereas at baseline IL-6 levels were related to both decreased and increased thickness of different cortical areas [84]. Increases in a peripheral inflammation composite score, based among others on IL-6, were also related to lower functional connectivity in particular in *APOE* ε4 carriers, independent of amyloid status [85, 86]. Moreover, a recent study found IL-6 to relate to higher amyloid burden (measured by PiB-PET) and future deposition in subjects with low hippocampal volume [87]. Due to the lack of interrelation with CSF levels, these findings are likely due to systemic inflammatory changes, rather than due to CNS neuroinflammation that transmits to the periphery. The mechanistic link between this relation is currently unknown, although it can be speculated that various physiologic, medical, environmental, or lifestyle factors could impact on both brain structure and systemic inflammation.

Finally, we found YKL-40 to correlate to its CSF levels as well as CSF neurodegeneration markers and structural MRI, but not to cognitive performance, clinical staging, or CSF biomarker-based schematics. YKL-40 can act as an acute phase protein with a range of functions in the regulation of proliferation, migration, cellular adhesion, and differentiation and is released by a plethora of cells [32]: In the periphery, YKL-40 is expressed under physiological conditions by metamyelocytes, granulocytes, macrophages, vascular smooth muscle cells, and synovial cells and under pathological conditions by various neoplasms as well as in fibrotic areas in liver diseases or during hepatitis C infection [88]. The liver also has the highest YKL-40 RNA levels compared to other tissues (Additional file 1: AF5). Additional to cancer, blood YKL-40 levels are elevated in diverse acute and chronic inflammatory disorders and in response to bacterial infections [89, 90]. In contrast, CSF levels of YKL-40 are considered markers of astroglia activation. The exact functions of YKL-40 in physiology and disease are not fully understood, though it is assumed to play a role in tissue remodeling, regulation of proliferation, adhesion, migration, differentiation, and protection of the extracellular matrix after stimulation of its expressing cells by inflammatory cytokines [32, 91]. Given the correlations between serum YKL-40 to CSF YKL-40 and CSF neurodegeneration marker levels on the one hand and structural MRI on the other, different scenarios are possible: First, YKL-40 might be transported from the CSF to serum, mixing with systemic levels of this protein, and thus partly reflecting astroglial activation induced by neurodegeneration. Furthermore, as the correlation itself does not provide information on the direction of YKL-40 exchange, it might just as well be the case that CSF levels are to some extent derived from peripheral protein levels that passed the blood–brain barrier. Other putative mechanisms could include the transfer of cytokines released during AD-related neuroinflammation to the periphery, stimulating peripheral YKL-40 expressing cells, as well the possibility that peripheral YKL-40 reflects systematic inflammatory processes that relate in an indirect manner to pathogenic developments in the brain. Currently, it remains unknown if there are any differences between YKL-40 derived from astrocytes compared to peripheral cells—such as specific post-translational modifications—that could be utilized to differentiate the origin of peripheral YKL-40. Nonetheless, our findings indicate that it constitutes one of the most promising markers for follow-up studies of serum samples of AD patients and the respective preceding stages. If handled and stored properly, YKL-40 is stable in serum for years, with serum levels higher, but nonetheless strongly correlated to plasma, enabling comparison between cohorts and most importantly longitudinal assessments [88].

In summary, our study shows that inflammation-related biomarkers in serum relate to AD neuropathology but have to be interpreted concerning their biology, correlating only in part to their corresponding CSF protein levels. In particular, our findings identified three targets for further evaluation: IL-6 as a proxy of systemic inflammation negatively related to the brain structure, sAXL relating to the biomarker-positive AD stage, and YKL-40 as the marker with the most pronounced relation to CSF biomarkers and structure.

### Limitations

The results presented in this manuscript have some limitations. Our approach for analysis was exploratory, accepting unadjusted *p* values in statistics to identify the most promising candidate markers for future, confirmatory follow-up analyses. As the sample size of our dataset, especially when trying to stratify sub-group populations, was limited, this approach can lead to power limitations resulting in potential false-positive, but also false-negative, results in significance testing. To address this limitation, we report exact *p* values and correlation strength whenever possible to enable identification of both the findings with the strongest effect size, as well as those where a minor effect might not have been identified due to power limitations. Furthermore, we focused our discussion on those markers with most consistent effects within the different models (e.g., observed to correlate to several Braak ROI) or across different analyses (e.g., between group-wise comparisons and structural MRI analysis). Our rationale for this selection was that such effects are less likely to derive from artifacts in the data, in contrast to spurious, “single hit” findings occurring in multiple testing analyzes.

Some of our findings are, to our knowledge, in line with previous reports—e.g., results observed for serum FABP-3, neurogranin, or YKL-40, but also the high serum to CSF correlation of CRP—which supports that our models have validity in general. However, many effects observed were of weak to modest strength, and further large-scale analyses will be required to verify if such effects hold true for the markers that have been less frequently tested in blood so far.

## Conclusions

In this study, we explored a panel of experimental inflammation-related biomarkers established in CSF for application as blood-based biomarkers. We conclude that some of these markers, such as sAXL, IL-6, and YKL-40, have potential as correlates of different AD features. However, due to each protein’s specific biology, observed effects do not translate one to one from CSF to blood, but rather manifest in periphery-specific patterns that require further exploration.

## Supplementary Information

**Additional file 1.** 

## Data Availability

The datasets generated and/or analyzed during the current study are not publicly available due to restrictions of the DELCOSE study but are available from the DELCODE study committee on reasonable request. For details and contact, please see https://www.dzne.de/en/research/studies/clinical-studies/delcode/.

## Abbreviations

Aβ: Amyloid beta
AD: Alzheimer’s disease
CI: 95% confidence interval
CRP: C-reactive protein
CSF: Cerebrospinal fluid
CV: Coefficient of variance
DAT: Dementia of the Alzheimer Type
DELCODE: DZNE—Longitudinal Cognitive Impairment and Dementia Study
DZNE: Deutsches Zentrum für Neurodegenerative Erkrankungen (German Center for Neurodegenerative Diseases)
FABP-3: Fatty acid binding protein 3
FCSRT: Free and Cued Selective Reminding Test
HC: Healthy controls
IL-6: Interleukin-6
Il-18: Interleukin-18
MCI: Mild cognitive impairment
MIF: Macrophage migration inhibitory factor
MMSE: Mini-Mental State Exam
MRI: Magnetic resonance imaging
Nf-L: Neurofilament light chain
PACC5: Preclinical Alzheimer’s Cognitive Composite 5
PET: Positron emission tomography
ROI: Region of interest
SCD: Subjective cognitive decline
SDMT: Symbol Digit Modalities Test
p-tau-181: Phospho-tau 181
t-tau: Total tau protein
TREM2: Triggering receptor expressed on myeloid cells 2

## References

[1] Ashton NJ, Leuzy A, Karikari TK, Mattsson-Carlgren N, Dodich A, Boccardi M, et al. The validation status of blood biomarkers of amyloid and phospho-tau assessed with the 5-phase development framework for AD biomarkers. Eur J Nucl Med Mol Imaging. 2021. [cited 2021 Mar 30]. Available from: http://link.springer.com/10.1007/s00259-021-05253-y. 10.1007/s00259-021-05253-y PMC8175325 33677733

[2] Heneka MT, Carson MJ, El Khoury J, Landreth GE, Brosseron F, Feinstein DL (2015). Neuroinflammation in Alzheimer’s disease. Lancet Neurol.

[3] Lai KSP, Liu CS, Rau A, Lanctôt KL, Köhler CA, Pakosh M (2017). Peripheral inflammatory markers in Alzheimer’s disease: a systematic review and meta-analysis of 175 studies. J Neurol Neurosurg Psychiatry.

[4] Brosseron F, Krauthausen M, Kummer M, Heneka MT. Body fluid cytokine levels in mild cognitive impairment and Alzheimer’s disease: a comparative overview. Mol Neurobiol. 2014; Available from: http://www.ncbi.nlm.nih.gov/pubmed/24567119. 10.1007/s12035-014-8657-1 PMC4182618 24567119

[5] Jessen F, Spottke A, Boecker H, Brosseron F, Buerger K, Catak C (2018). Design and first baseline data of the DZNE multicenter observational study on predementia Alzheimer’s disease (DELCODE). Alzheimers Res Ther.

[6] Brosseron F, Kleemann K, Kolbe CC, Santarelli F, Castro-Gomez S, Tacik P, et al. Interrelations of Alzheimer’s disease candidate biomarkers neurogranin, fatty acid-binding protein 3 and ferritin to neurodegeneration and neuroinflammation. J Neurochem. 2020;157:2210-24. 10.1111/jnc.15175 32894885

[7] Brosseron F, Kolbe CC, Santarelli F, Carvalho S, Antonell A, Castro-Gomez S, et al. Multicenter Alzheimer’s and Parkinson’s disease immune biomarker verification study. Alzheimers Dement J Alzheimers Assoc. 2019;16:292-304. 10.1016/j.jalz.2019.07.018 31630996

[8] Brosseron F, Traschütz A, Widmann CN, Kummer MP, Tacik P, Santarelli F (2018). Characterization and clinical use of inflammatory cerebrospinal fluid protein markers in Alzheimer’s disease. Alzheimers Res Ther.

[9] Brosseron F, Maass A, Kleineidam L, Ravichandran KA, González PG, McManus RM, et al. Soluble TAM receptors sAXL and sTyro3 predict structural and functional protection in Alzheimer’s disease. Neuron. 2021;110:1009-1022.e4. 10.1016/j.neuron.2021.12.016 34995486

[10] Oikonomidi A, Tautvydaitė D, Gholamrezaee MM, Henry H, Bacher M, Popp J (2017). Macrophage migration inhibitory factor is associated with biomarkers of Alzheimer’s disease pathology and predicts cognitive decline in mild cognitive impairment and mild dementia. J Alzheimers Dis JAD.

[11] Kiddle SJ, Thambisetty M, Simmons A, Riddoch-Contreras J, Hye A, Westman E, et al. Plasma based markers of [11C] PiB-PET brain amyloid burden. PLoS ONE. 2012;7(9) Available from: https://www.ncbi.nlm.nih.gov/pmc/articles/PMC3454385/. [cited 2020 Dec 21]. 10.1371/journal.pone.0044260 PMC3454385 23028511

[12] Morgan AR, Touchard S, Leckey C, O’Hagan C, Nevado-Holgado AJ, Barkhof F (2019). Inflammatory biomarkers in Alzheimer’s disease plasma. Alzheimers Dement.

[13] Zabel M, Schrag M, Mueller C, Zhou W, Crofton A, Petersen F (2012). Assessing candidate serum biomarkers for Alzheimer’s disease: a longitudinal study. J Alzheimers Dis JAD.

[14] Chen M, Xia W (2020). Proteomic profiling of plasma and brain tissue from Alzheimer’s disease patients reveals candidate network of plasma biomarkers. J Alzheimers Dis JAD.

[15] Gezen-Ak D, Dursun E, Hanağası H, Bilgiç B, Lohman E, Araz ÖS (2013). BDNF, TNFα, HSP90, CFH, and IL-10 serum levels in patients with early or late onset Alzheimer’s disease or mild cognitive impairment. J Alzheimers Dis JAD.

[16] Hye A, Lynham S, Thambisetty M, Causevic M, Campbell J, Byers HL (2006). Proteome-based plasma biomarkers for Alzheimer’s disease. Brain..

[17] Thambisetty M, Hye A, Foy C, Daly E, Glover A, Cooper A (2008). Proteome-based identification of plasma proteins associated with hippocampal metabolism in early Alzheimer’s disease. J Neurol.

[18] Akuffo EL, Davis JB, Fox SM, Gloger IS, Hosford D, Kinsey EE (2008). The discovery and early validation of novel plasma biomarkers in mild-to-moderate Alzheimer’s disease patients responding to treatment with rosiglitazone. Biomarkers..

[19] Bennett S, Grant M, Creese AJ, Mangialasche F, Cecchetti R, Cooper HJ (2012). Plasma levels of complement 4a protein are increased in Alzheimer’s disease. Alzheimer Dis Assoc Disord.

[20] Song F, Poljak A, Kochan NA, Raftery M, Brodaty H, Smythe GA, et al. Plasma protein profiling of mild cognitive impairment and Alzheimer’s disease using iTRAQ quantitative proteomics. Proteome Sci. 2014; [cited 2021 Mar 2];12(1):5. Available from: http://proteomesci.biomedcentral.com/articles/10.1186/1477-5956-12-5. 10.1186/1477-5956-12-5 PMC3898732 24433274

[21] Muenchhoff J, Poljak A, Song F, Raftery M, Brodaty H, Duncan M (2015). Plasma protein profiling of mild cognitive impairment and Alzheimer’s disease across two independent cohorts. J Alzheimers Dis JAD.

[22] Ashton NJ, Kiddle SJ, Graf J, Ward M, Baird AL, Hye A (2015). Blood protein predictors of brain amyloid for enrichment in clinical trials?. Alzheimers Dement Diagn Assess Dis Monit.

[23] Williams MA, Haughton D, Stevenson M, Craig D, Passmore AP, Silvestri G (2015). Plasma complement factor H in Alzheimer’s disease. J Alzheimers Dis JAD.

[24] Sattlecker M, Khondoker M, Proitsi P, Williams S, Soininen H, Kłoszewska I (2016). Longitudinal protein changes in blood plasma associated with the rate of cognitive decline in Alzheimer’s disease. J Alzheimers Dis.

[25] Cheng Z, Yin J, Yuan H, Jin C, Zhang F, Wang Z, et al. Blood-derived plasma protein biomarkers for Alzheimer’s disease in Han Chinese. Front Aging Neurosci. 2018;10 Available from: https://www.ncbi.nlm.nih.gov/pmc/articles/PMC6305130/. [cited 2021 Mar 2]. 10.3389/fnagi.2018.00414 PMC6305130 30618720

[26] Westwood S, Baird AL, Hye A, Ashton NJ, Nevado-Holgado AJ, Anand SN (2018). Plasma protein biomarkers for the prediction of CSF amyloid and tau and [18F]-Flutemetamol PET scan result. Front Aging Neurosci.

[27] Morgan AR, Touchard S, O’Hagan C, Sims R, Majounie E, Escott-Price V (2017). The correlation between inflammatory biomarkers and polygenic risk score in Alzheimer’s disease. J Alzheimers Dis JAD.

[28] Westwood S, Leoni E, Hye A, Lynham S, Khondoker MR, Ashton NJ (2016). Blood-based biomarker candidates of cerebral amyloid using PiB PET in non-demented elderly. J Alzheimers Dis JAD.

[29] Ohara T, Hata J, Tanaka M, Honda T, Yamakage H, Yoshida D (2019). Serum soluble triggering receptor expressed on myeloid cells 2 as a biomarker for incident dementia: the Hisayama study. Ann Neurol.

[30] Hu N, Tan MS, Yu JT, Sun L, Tan L, Wang YL (2013). Increased expression of TREM2 in peripheral blood of Alzheimer’s disease patients. J Alzheimers Dis.

[31] Liu D, Cao B, Zhao Y, Huang H, McIntyre RS, Rosenblat JD, et al. Soluble TREM2 changes during the clinical course of Alzheimer’s disease: a meta-analysis. Neurosci Lett. 2018;(686):10–6. 10.1016/j.neulet.2018.08.038 30171911

[32] Wilczyńska K, Waszkiewicz N. Diagnostic utility of selected serum dementia biomarkers: amyloid β-40, amyloid β-42, tau protein, and YKL-40: a review. J Clin Med. 2020;9(11) Available from: https://www.ncbi.nlm.nih.gov/pmc/articles/PMC7692800/. [cited 2021 Feb 22]. 10.3390/jcm9113452 PMC7692800 33121040

[33] Craig-Schapiro R, Perrin RJ, Roe CM, Xiong C, Carter D, Cairns NJ (2010). YKL-40: a novel prognostic fluid biomarker for preclinical Alzheimer’s disease. Biol Psychiatry.

[34] Grewal R, Haghighi M, Huang S, Smith AG, Cao C, Lin X (2016). Identifying biomarkers of dementia prevalent among amnestic mild cognitively impaired ethnic female patients. Alzheimers Res Ther.

[35] Vergallo A, Lista S, Lemercier P, Chiesa PA, Zetterberg H, Blennow K (2020). Association of plasma YKL-40 with brain amyloid-β levels, memory performance, and sex in subjective memory complainers. Neurobiol Aging.

[36] Wolfsgruber S, Kleineidam L, Weyrauch AS, Barkhoff M, Röske S, Peters O (2022). Relevance of subjective cognitive decline in older adults with a first-degree family history of Alzheimer’s disease. J Alzheimers Dis JAD.

[37] Suarez-Calvet M, Kleinberger G, Araque Caballero MA, Brendel M, Rominger A, Alcolea D (2016). sTREM2 cerebrospinal fluid levels are a potential biomarker for microglia activity in early-stage Alzheimer’s disease and associate with neuronal injury markers. EMBO Mol Med.

[38] Bertens D, Tijms BM, Scheltens P, Teunissen CE, Visser PJ (2017). Unbiased estimates of cerebrospinal fluid β-amyloid 1-42 cutoffs in a large memory clinic population. Alzheimers Res Ther.

[39] Fischl B, Salat DH, Busa E, Albert M, Dieterich M, Haselgrove C (2002). Whole brain segmentation: automated labeling of neuroanatomical structures in the human brain. Neuron..

[40] Fischl B, van der Kouwe A, Destrieux C, Halgren E, Ségonne F, Salat DH (2004). Automatically parcellating the human cerebral cortex. Cereb Cortex N Y N 1991.

[41] Schöll M, Lockhart SN, Schonhaut DR, O’Neil JP, Janabi M, Ossenkoppele R (2016). PET imaging of tau deposition in the aging human brain. Neuron..

[42] Baker SL, Maass A, Jagust WJ (2017). Considerations and code for partial volume correcting [18F]-AV-1451 tau PET data. Data Brief.

[43] Braak H, Braak E (1991). Neuropathological stageing of Alzheimer-related changes. Acta Neuropathol (Berl).

[44] Papp KV, Rentz DM, Orlovsky I, Sperling RA, Mormino EC (2017). Optimizing the preclinical Alzheimer’s cognitive composite with semantic processing: the PACC5. Alzheimers Dement N Y N.

[45] Proust-Lima C, Dartigues JF, Jacqmin-Gadda H (2011). Misuse of the linear mixed model when evaluating risk factors of cognitive decline. Am J Epidemiol.

[46] Proust-Lima C, Philipps V, Liquet B. Estimation of extended mixed models using latent classes and latent processes: the R package lcmm. J Stat Softw. 2017;78(1, 1):–56 Available from: https://www.jstatsoft.org/index.php/jss/article/view/v078i02. [cited 2021 Jan 21].

[47] Uhlén M, Björling E, Agaton C, Szigyarto CAK, Amini B, Andersen E (2005). A human protein atlas for normal and cancer tissues based on antibody proteomics. Mol Cell Proteomics MCP.

[48] Uhlén M, Fagerberg L, Hallström BM, Lindskog C, Oksvold P, Mardinoglu A (2015). Proteomics. Tissue-based map of the human proteome. Science..

[49] Uhlen M, Karlsson MJ, Zhong W, Tebani A, Pou C, Mikes J (2019). A genome-wide transcriptomic analysis of protein-coding genes in human blood cells. Science..

[50] Sjöstedt E, Zhong W, Fagerberg L, Karlsson M, Mitsios N, Adori C (2020). An atlas of the protein-coding genes in the human, pig, and mouse brain. Science..

[51] Karlsson M, Zhang C, Méar L, Zhong W, Digre A, Katona B (2021). A single-cell type transcriptomics map of human tissues. Sci Adv.

[52] Jack CR, Bennett DA, Blennow K, Carrillo MC, Feldman HH, Frisoni GB (2016). A/T/N: an unbiased descriptive classification scheme for Alzheimer disease biomarkers. Neurology..

[53] Mollenhauer B, Steinacker P, Bahn E, Bibl M, Brechlin P, Schlossmacher MG (2007). Serum heart-type fatty acid-binding protein and cerebrospinal fluid tau: marker candidates for dementia with Lewy bodies. Neurodegener Dis.

[54] Teunissen CE, Veerhuis R, De Vente J, Verhey FRJ, Vreeling F, van Boxtel MPJ (2011). Brain-specific fatty acid-binding protein is elevated in serum of patients with dementia-related diseases. Eur J Neurol.

[55] Wada-Isoe K, Imamura K, Kitamaya M, Kowa H, Nakashima K (2008). Serum heart-fatty acid binding protein levels in patients with Lewy body disease. J Neurol Sci.

[56] Thumser AE, Moore JB, Plant NJ (2014). Fatty acid binding proteins: tissue-specific functions in health and disease. Curr Opin Clin Nutr Metab Care.

[57] Gangishetti U, Christina Howell J, Perrin RJ, Louneva N, Watts KD, Kollhoff A (2018). Non-beta-amyloid/tau cerebrospinal fluid markers inform staging and progression in Alzheimer’s disease. Alzheimers Res Ther.

[58] Chiasserini D, Biscetti L, Eusebi P, Salvadori N, Frattini G, Simoni S (2017). Differential role of CSF fatty acid binding protein 3, α-synuclein, and Alzheimer’s disease core biomarkers in Lewy body disorders and Alzheimer’s dementia. Alzheimers Res Ther.

[59] Rankin EB, Giaccia AJ (2016). The receptor tyrosine kinase AXL in cancer progression. Cancers.

[60] Smirne C, Rigamonti C, De Benedittis C, Sainaghi PP, Bellan M, Burlone ME (2019). Gas6/TAM signaling components as novel biomarkers of liver fibrosis. Dis Markers.

[61] Pagani S, Bellan M, Mauro D, Castello LM, Avanzi GC, Lewis MJ (2020). New insights into the role of Tyro3, Axl, and Mer receptors in rheumatoid arthritis. Dis Markers.

[62] Tondo G, Perani D, Comi C (2019). TAM receptor pathways at the crossroads of neuroinflammation and neurodegeneration. Dis Markers.

[63] DuBois JC, Ray AK, Davies P, Shafit-Zagardo B (2020). Anti-Axl antibody treatment reduces the severity of experimental autoimmune encephalomyelitis. J Neuroinflammation.

[64] Zhao W, Fan J, Kulic I, Koh C, Clark A, Meuller J (2020). Axl receptor tyrosine kinase is a regulator of apolipoprotein E. Mol Brain.

[65] Shafit-Zagardo B, Gruber RC, DuBois JC (2018). The role of TAM family receptors and ligands in the nervous system: From development to pathobiology. Pharmacol Ther.

[66] Mattsson N, Insel P, Nosheny R, Zetterberg H, Trojanowski JQ, Shaw LM (2013). CSF protein biomarkers predicting longitudinal reduction of CSF β-amyloid42 in cognitively healthy elders. Transl Psychiatry.

[67] Kang S, Narazaki M, Metwally H, Kishimoto T (2020). Historical overview of the interleukin-6 family cytokine. J Exp Med.

[68] Hirano T (2021). IL-6 in inflammation, autoimmunity and cancer. Int Immunol.

[69] Niculet E, Chioncel V, Elisei AM, Miulescu M, Buzia OD, Nwabudike LC, et al. Multifactorial expression of IL-6 with update on COVID-19 and the therapeutic strategies of its blockade (Review). Exp Ther Med. 2021;21(3) Available from: https://www.ncbi.nlm.nih.gov/pmc/articles/PMC7851683/. [cited 2021 Mar 24]. 10.3892/etm.2021.9693 PMC7851683 33603870

[70] Hazen J, Vistnes M, Barca ML, Eldholm RS, Persson K, Brækhus A (2020). The association between circulating inflammatory markers and the progression of Alzheimer disease in Norwegian memory clinic patients with mild cognitive impairment or dementia. Alzheimer Dis Assoc Disord.

[71] Cisbani G, Koppel A, Knezevic D, Suridjan I, Mizrahi R, Bazinet RP (2020). Peripheral cytokine and fatty acid associations with neuroinflammation in AD and aMCI patients: an exploratory study. Brain Behav Immun.

[72] Boots EA, Castellanos KJ, Zhan L, Barnes LL, Tussing-Humphreys L, Deoni SCL, et al. Inflammation, cognition, and white matter in older adults: an examination by race. Front Aging Neurosci. 2020;12 Available from: https://www.ncbi.nlm.nih.gov/pmc/articles/PMC7662133/. [cited 2021 Apr 1]. 10.3389/fnagi.2020.553998 PMC7662133 33192454

[73] Marsland AL, Gianaros PJ, Kuan DCH, Sheu LK, Krajina K, Manuck SB (2015). Brain morphology links systemic inflammation to cognitive function in midlife adults. Brain Behav Immun.

[74] Jefferson AL, Massaro JM, Wolf PA, Seshadri S, Au R, Vasan RS (2007). Inflammatory biomarkers are associated with total brain volume: the Framingham Heart Study. Neurology..

[75] Schmidt MF, Freeman KB, Windham BG, Griswold ME, Kullo IJ, Turner ST (2016). Associations between serum inflammatory markers and hippocampal volume in a community sample. J Am Geriatr Soc.

[76] O’Donovan A, Chao LL, Paulson J, Samuelson KW, Shigenaga JK, Grunfeld C (2015). Altered inflammatory activity associated with reduced hippocampal volume and more severe posttraumatic stress symptoms in Gulf War veterans. Psychoneuroendocrinology..

[77] Aribisala BS, Wiseman S, Morris Z, Valdés-Hernández MC, Royle NA, Maniega SM (2014). Circulating inflammatory markers are associated with magnetic resonance imaging-visible perivascular spaces but not directly with white matter hyperintensities. Stroke..

[78] Kakeda S, Watanabe K, Katsuki A, Sugimoto K, Igata N, Ueda I (2018). Relationship between interleukin (IL)-6 and brain morphology in drug-naïve, first-episode major depressive disorder using surface-based morphometry. Sci Rep.

[79] Satizabal CL, Zhu YC, Mazoyer B, Dufouil C, Tzourio C (2012). Circulating IL-6 and CRP are associated with MRI findings in the elderly: the 3C-Dijon Study. Neurology..

[80] Gu Y, Manly JJ, Mayeux RP, Brickman AM (2018). An inflammation-related nutrient pattern is associated with both brain and cognitive measures in a multiethnic elderly population. Curr Alzheimer Res.

[81] Gu Y, Vorburger R, Scarmeas N, Luchsinger JA, Manly JJ, Schupf N (2017). Circulating inflammatory biomarkers in relation to brain structural measurements in a non-demented elderly population. Brain Behav Immun.

[82] Satizabal CL, Zhu YC, Dufouil C, Tzourio C (2013). Inflammatory proteins and the severity of dilated Virchow-Robin Spaces in the elderly. J Alzheimers Dis JAD.

[83] Ironside M, Admon R, Maddox SA, Mehta M, Douglas S, Olson DP (2020). Inflammation and depressive phenotypes: evidence from medical records from over 12 000 patients and brain morphology. Psychol Med.

[84] McCarrey AC, Pacheco J, Carlson OD, Egan JM, Thambisetty M, An Y (2014). Interleukin-6 is linked to longitudinal rates of cortical thinning in aging. Transl Neurosci.

[85] Walker KA, Gross AL, Moghekar AR, Soldan A, Pettigrew C, Hou X (2020). Association of peripheral inflammatory markers with connectivity in large-scale functional brain networks of non-demented older adults. Brain Behav Immun.

[86] Nusslock R, Brody G, Armstrong C, Carroll A, Sweet LH, Yu T (2019). Higher peripheral inflammatory signaling associated with lower resting state functional brain connectivity in emotion regulation and central executive networks. Biol Psychiatry.

[87] Oberlin LE, Erickson KI, Mackey R, Klunk WE, Aizenstein H, Lopresti BJ (2021). Peripheral inflammatory biomarkers predict the deposition and progression of amyloid-β in cognitively unimpaired older adults. Brain Behav Immun.

[88] Johansen JS, Jensen BV, Roslind A, Nielsen D, Price PA (2006). Serum YKL-40, a new prognostic biomarker in cancer patients?. Cancer Epidemiol Biomark Prev Publ Am Assoc Cancer Res Cosponsored Am Soc Prev Oncol.

[89] Tong X, Wang D, Liu S, Ma Y, Li Z, Tian P (2018). The YKL-40 protein is a potential biomarker for COPD: a meta-analysis and systematic review. Int J Chron Obstruct Pulmon Dis.

[90] Lee CG, Da Silva CA, Dela Cruz CS, Ahangari F, Ma B, Kang MJ (2011). Role of chitin and chitinase/chitinase-like proteins in inflammation, tissue remodeling, and injury. Annu Rev Physiol.

[91] Muszyński P, Groblewska M, Kulczyńska-Przybik A, Kułakowska A, Mroczko B (2017). YKL-40 as a potential biomarker and a possible target in therapeutic strategies of Alzheimer’s disease. Curr Neuropharmacol.

