# Supplementary material for: Serum IL-6, sAXL, and YKL-40 as systemic correlates of reduced brain structure and function in Alzheimer’s disease: results from the DELCODE study
Source: Alzheimers Res Ther. 2023 Jan 12;15:13. doi: 10.1186/s13195-022-01118-0 (PMC9835320; doi:10.1186/s13195-022-01118-0)

**Additional File 1 Tables & Figures**

Serum IL-6, sAXL and YKL-40 as systemic correlates of reduced brain structure in the DELCODE study

| **Contents** | | **Page** |
| --- | --- | --- |
|  | |  |
| **AT 1** | Biomarker covariates | 2 |
| **AF 1** | Serum to CSF correlation matrix alternate models | 3 |
| **AT 2** | Biomarkers by screening diagnosis | 5 |
| **AT 3** | Biomarkers by A/T scheme | 8 |
| **AT 4** | Biomarkers by A/N scheme | 11 |
| **AT 5** | Biomarkers by AD Spectrum scheme | 14 |
| **AF 2** | Braak ROI alternate models | 17 |
| **AT 6** | PACC5 Score analysis | 18 |
| **AT 7** | PACC5 Score analysis alternate models | 19 |
| **AF 3** | Human Protein Atlas: Brain RNA expression | 20 |
| **AF 4** | Human Protein Atlas: Blood RNA expression | 21 |
| **AF 5** | Human Protein Atlas: Tissue RNA expression | 22 |
| **AF 6** | Human Protein Atlas: Protein Expression Levels | 23 |

**Additional Table 1: Biomarker covariates**

The table displays relations of the differentially distributed demographic covariates to the analyzed biomarkers. The cohort was dichotomized by either sex or *APOE* status and biomarker levels were correlated to age and BMI (Mann-Whitney U test for categorical, spearman non-parametric correlation for steady variables; significance level α = 0.05, Bonferroni-adjusted (adjusted for 20 tested markers) α = 0.0025). For each tested biomarker, p value and fold change or spearman r values are displayed. Covariates that reached α are highlighted in *italics*, those reaching the Bonferroni-adjusted α are highlighted **bold**. Nearly all tested biomarkers showed relations to one or more covariates. By covariate, the strongest observed effects on serum levels were: Ferritin increased 1.5x in female subjects; ApoE reduced 0.6x in ε4 carriers; age-dependent increase of IL-6 (r = 0.280); BMI-dependent increase in complement factor H (r = 0.305). All 4 variables were used for adjustment in all analyzes performed.

| **Protein** | **Sex**  (p / fold change in female) | ***APOE***  (p / fold change in ε4 carriers) | **Age**  (p / r) | **BMI**  (p / r) | **Covariates** |
| --- | --- | --- | --- | --- | --- |
| sTREM2 | 0.983 | 0.604 | 0.120 / 0.089 | 0.305 / 0.059 | **-** |
| sAXL | 0.294 | *0.004 / 1.07x* | 0.446 / 0.044 | 0.445 / 0.044 | *APOE* |
| sTyro3 | 0.592 | 0.280 | 0.152 / -0.082 | 0.036 / 0.120 | - |
| CRP | 0.735 | *0.009 / 0.76x* | 0.996 / 0.000 | **8x10E-5 / 0.223** | *APOE*, **BMI** |
| YKL-40 | **8x10E-4 / 1.25x** | 0.548 | **6x10E-5 / 0.227** | *0.013 / 0.142* | **Sex**, **Age**, BMI |
| IL-6 | **0.002 / 1.25x** | 0.543 | **6x10E-7 / 0.280** | *0.006 / 0.156* | **Sex, Age**, BMI |
| Il-18 | **2x10E-7 / 1.28x** | 0.292 | 0.839 / 0.012 | *0.050 / 0.112* | **Sex**, BMI |
| CXCL10 | 0.557 | 0.714 | *0.017 / 0.136* | 0.421 / -0.046 | Age |
| CCL2 | 0.337 | 0.478 | 0.990 / -0.001 | 0.357 / 0.053 | - |
| MIF | *0.011 / 1.14x* | 0.185 | 0.552 / 0.034 | 0.751 / -0.018 | Sex |
| C1q | 0.198 | *0.017 / 1.05x* | 0.514 / -0.037 | 0.209 / 0.072 | *APOE* |
| C3 | 0.782 | 0.692 | 0.940 / -0.004 | **0.002 / 0.175** | **BMI** |
| C3b | *0.027 / 0.85x* | 0.328 | 0.635 / -0.029 | 0.340 / -0.058 | Sex |
| C4 | *0.030 / 0.96x* | 0.302 | 0.817 / 0.013 | 0.016 / 0.138 | Sex |
| Factor B | *0.006 / 0.95x* | 0.414 | 0.082 / -0.099 | *0.032 / 0.123* | Sex, BMI |
| Factor H | 0.192 | 0.591 | 0.482 / -0.040 | **5x10E-8 / 0.305** | **BMI** |
| FABP-3 | **3x10E-6 / 1.20x** | 0.346 | **8x10E-7 / 0.277** | 0.257 / 0.065 | **Sex**, **Age** |
| Neurogranin | 0.872 | *0.050 / 1.14x* | 0.774 / -0.016 | 0.885 / -0.010 | *APOE* |
| Ferritin | **3x10E-6 / 1.51** | 0.772 | 0.432 / -0.045 | **2x10E-5 / 0.241** | **Sex, BMI** |
| ApoE | **4x10E-4 / 0.85x** | **<1x10E-15 / 0.60x** | 0.206 / -0.072 | 0.978 / 0.002 | **Sex, *APOE*** |

**Additional Figure 1: Serum to CSF correlation matrix alternate models**

Figure displayed on next page. Non-parametric Spearman correlation matrix calculated for serum biomarker levels against their CSF counterparts and CSF AD / neurodegeneration markers. Spearman r is displayed left with stronger with significant correlations colored red (positive) or blue (negative). Spearman p is displayed on the right with darker blue indicating stronger significance. All models were adjusted for age, sex, *APOE* status and BMI, comparable to manuscript figure 1. **A)** Exclusion of MCI and AD subjects from the analysis. There was little effect on the serum to CSF correlations of the markers themselves; while FABP-3 and YKL-40 did not correlate to other neurodegeneration markers, like neurogranin, anymore. **B)** Additional adjustment of correlations for CSF levels of Aβ42/40 ratio and p-tau-181 as proxies of AD pathology. There was little effect on the serum to CSF relations of inflammatory markers, but FABP-3 and YKL-40 lost the correlations to CSF neurodegeneration markers observed without adjustment for pathology. Correlations to plasma Nf-L were barely affected between the different models.

**
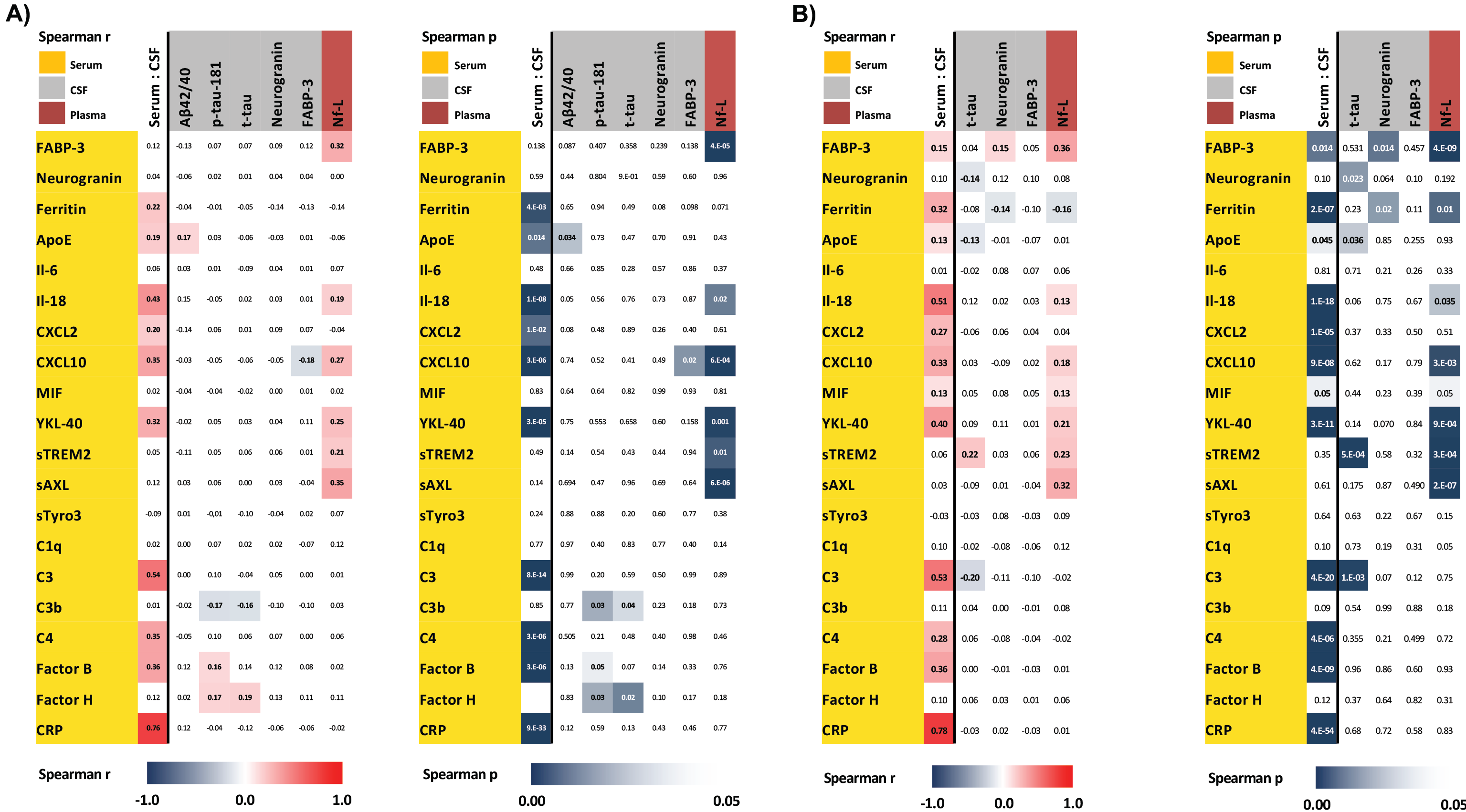
**

**Additional Table 2: Biomarkers by screening diagnosis**

Experimental biomarkers used in this study are described between screening diagnosis groups of subjects by median ± standard deviation, minimum and maximum value. Differences between subject groups were tested by non-parametric Kruskal-Wallis test including pairwise comparisons adjusted for multiple testing, followed by ANCOVA using log-transformed protein level values to approximate normal distribution. In ANCOVA, first sensitivity assessment for covariates was performed including all 4 covariates (age, sex, *APOE* status, BMI), followed by a final model including only the significant covariates. Covariates are reported with their ANCOVA significance test result. ANCOVA p is reported for the covariate adjusted group comparison. Markers are highlighted *italic* if they reached α = 0.05 (sTREM2, CRP, IL-6 and neurogranin), and **bold** if they reached Bonferroni-adjusted α = 0.0025 (only FABP-3). For pairwise comparisons, α was set at 0.05 as pairwise tests were multiplicity-adjusted already in the Kruskal Wallis test. “None after adjustment” refers to pairwise test insignificant after multiplicity adjustment or markers insignificant after covariate adjustment.

| **Marker** | **HC** | **Relatives** | **SCD** | **MCI** | **AD** | **Kruskal-**  **Wallis** | **Covariates** | **ANCOVA p** |
| --- | --- | --- | --- | --- | --- | --- | --- | --- |
| N | 74 | 23 | 99 | 75 | 38 |  |  |  |
|  |  |  |  |  |  |  |  |  |
| sTREM2  (ng/ml) | 7.4 ± 9.1,  1.6 – 16.4 | 13.9 ± 13.6,  2.2 – 69.4 | 7.9 ± 17.6,  2.2 – 14.0 | 9.0 ± 16.9,  2.3 – 76.0 | 8.3 ± 17.5,  1.9 – 75.6 | KW p = 0.244 | - | - |
| **sAXL**  **(ng/ml)** | 41.1 ± 7.8,  22.1 – 65.7 | 42.6 ± 6.4,  34.3 – 53.2 | 40.7 ± 8.8,  23.8 – 87.3 | 44.5 ± 12.4,  23.7 – 87.3 | 48.5 ± 9.8,  35.1 – 83.5 | KW p = 3x10E-4  HC Vs AD = 0.001  SCD Vs AD = 0.001 | - | - |
| sTyro3  (pg/ml) | 1523 ± 405,  547 - 3038 | 1492 ± 327,  923 - 2103 | 1445 ± 348,  764 - 3017 | 1519 ± 369,  626 - 2180 | 1679 ± 373,  1098 - 2488 | KW p = 0.133 | BMI (0.003) | 0.067 |
| *CRP*  *(µg/ml)* | 1.6 ± 4.6,  0.3 – 25.4 | 1.3 ± 2.4,  0.3 – 8.0 | 1.1 ± 2.5,  0.2 – 11.7 | 2.1 ± 11.1,  0.2 – 89.9 | 1.2 ± 7.6,  0.1 – 46.8 | KW p = 0.017  SCD VS. MCI = 0.035 | BMI (0.001)  APOE (0.038) | 0.016 |
| YKL-40  (ng/ml) | 83 ± 151,  23 - 1020 | 56 ± 86,  30 – 383 | 78 ± 332,  27 - 3178 | 111 ± 100,  17 - 488 | 138 ± 112,  22 - 514 | KW p 2x10E-4  None after adjustment | Age (0.002)  Sex (0.002) | 0.108 |
| IL-6  (pg/ml) | 1.7 ± 4.2,  0.4 – 34.0 | 1.3 ± 2.5,  0.5 – 12.2 | 1.6 ± 1.6,  0.6 – 10.2 | 2.1 ± 1.6,  0.9 – 8.7 | 1.8 ± 3.5,  0.7 – 19.8 | KW p = 0.021  None after adjustment | Age (2x10E-5)  BMI (0.018) | 0.491 |
| Il-18  (pg/ml) | 260 ± 124,  98 – 948 | 212 ± 85,  140 – 441 | 239 ± 120,  120 – 824 | 287 ± 146,  84 – 909 | 237 ± 214,  105 – 1222 | KW p = 0.124 | Sex (1x10E-6) | 0.634 |
| CXCL10  (pg/ml) | 343 ± 245,  97 - 1312 | 317 ± 155,  216 – 903 | 361 ± 981,  158 – 9973 | 341 ± 154,  110 – 1009 | 407 ± 185,  169 – 894 | KW p = 0.139 | Age (0.003) | 0.067 |
| CCL2  (pg/ml) | 353 ± 104,  213 - 698 | 330 ± 107,  182 - 625 | 343 ± 89,  212 – 797 | 360 ± 184,  215 – 1729 | 332 ± 103,  241 - 729 | KW p = 0.524 | - | - |
| MIF  (ng/ml) | 18.0 ± 11.3,  10.0 – 97.3 | 16.0 ± 38.0,  9.1 – 196.3 | 16.7 ± 8.8,  7.6 – 90.7 | 17.1 ± 15.3,  8.1 – 103.4 | 15.7 ± 11.5,  7.1 – 75.0 | KW p = 0.185 | - | - |
| C1q  (µg/ml) | 134 ± 25,  80 - 187 | 129 ± 20,  97 - 167 | 133 ± 42,  60 – 372 | 131 ± 30,  81 - 244 | 148 ± 29,  94 - 261 | KW p = 0.090 | APOE (0.030) | 0.323 |
| C3  (µg/ml) | 26.7 ± 18.2,  65.5 – 531.7 | 21.5 ± 33.8,  11.9 – 180.3 | 25.3 ± 42.9,  8.2 – 365.6 | 28.6 ± 36.2,  9.9 – 256.0 | 25.3 ± 24.8,  13.1 – 156.5 | KW p = 0.075 | BMI (0.034) | 0.202 |
| C3b  (µg/ml) | 218 ± 81,  66 - 532 | 185 ± 124,  43 - 643 | 181 ± 259,  89 - 2163 | 199 ± 213,  59 - 1548 | 240 ± 104,  107 - 541 | KW p = 0.086 | - | - |
| C4  (µg/ml) | 124 ± 23,  60 – 182 | 121 ± 18,  92 – 161 | 126 ± 24,  85 – 205 | 126 ± 32,  84 – 337 | 132 ± 60,  85 – 478 | KW p = 0.328 | BMI (0.019)  Sex (0.028) | 0.159 |
| Factor B  (µg/ml) | 244 ± 53,  145 - 386 | 250 ± 35,  192 - 334 | 244 ± 49,  149 - 482 | 247 ± 51,  167 - 407 | 244 ± 48,  168 - 370 | KW p = 0.910 | Sex (0.009)  BMI (0.008) | 0.691 |
| Factor H  (ng/ml) | 365 ± 59,  218 – 458 | 345 ± 52,  290 – 475 | 348 ± 63,  224 – 635 | 367 ± 59,  243 – 528 | 361 ± 43,  283 – 471 | KW p = 0.389 | Sex (0.013)  BMI (1x10E-8) | 0.418 |
| *FABP-3*  *(pg/ml)* | 5162 ± 1971,  1763 - 16430 | 4583 ± 1734,  3780 - 9700 | 5041 ± 2696,  1735 - 23078 | 5768 ± 3513,  2694 - 26560 | 6130 ± 2918,  3444 - 17753 | KW p = 0.005  SCD Vs AD = 0.029 | Age (4x10E-6)  Sex (2x10E-6) | 0.020 |
| Neurogranin  (pg/ml) | 283 ± 456,  77 - 3789 | 215 ± 131,  84 - 720 | 264 ± 175,  87 - 1400 | 242 ± 314,  74 - 2231 | 229 ± 140,  35 - 657 | KW p = 0.061 | - | - |
| Ferritin  (ng/ml) | 329 ± 209,  20 – 992 | 338 ± 181,  35 – 796 | 299 ± 206,  26 – 1031 | 309 ± 234,  31 – 980 | 239 ± 259,  17 – 972 | KW p = 0.371 | Sex (1x10E-4)  BMI (4x10E-4) | 0.160 |
| ApoE  (µg/ml) | 6.0 ± 3.4,  2.2 – 20.5 | 6.2 ± 2.4,  2.6 – 12.5 | 5.0 ± 2.1,  1.1 – 13.8 | 5.0 ± 4.4,  1.7 – 35.1 | 4.6 ± 2.3,  1.4 – 10.6 | KW p = 0.010  AD Vs HC = 0.020 | Sex (2x10E-4)  APOE (2x10E-24) | 0.304 |

**Additional Table 3: Biomarkers by A/T scheme**

Experimental biomarkers between A/T (Amyloid / p-tau-181) scheme groups of subjects by median ± standard deviation, minimum and maximum value. Differences between subject groups were tested by non-parametric Kruskal-Wallis test, followed by ANCOVA using log-transformed protein level values to approximate normal distribution. In ANCOVA, first sensitivity assessment for covariates was performed including all 4 covariates (age, sex, *APOE* status, BMI), followed by a final model including only the significant covariates. Covariates are reported with their ANCOVA significance test result. ANCOVA p is reported for the covariate adjusted group comparison. Markers are highlighted *italic* if they reached α = 0.05 (sAXL), and **bold** if they reached Bonferroni-adjusted α = 0.0025 (not reached by any marker after adjustment for covariates). For pairwise comparisons of single groups, all p values are adjusted for multiple testing with α set at 0.05.

| **Marker** | **A- T-** | **A- T+** | **A+ T-** | **A+ T+** | **Kruskal-**  **Wallis** | **Covariates** | **ANCOVA p** |
| --- | --- | --- | --- | --- | --- | --- | --- |
| N | 167 | 7 | 71 | 63 |  |  |  |
|  |  |  |  |  |  |  |  |
| sTREM2  (ng/ml) | 8.1 ± 11.1,  1.6 – 76.0 | 6.0 ± 21.1,  2.3 – 57.3 | 9.5 ± 21.1,  2.2 – 140.2 | 7.8 ± 16.9,  2.0 – 72.8 | KW p = 0.597 | - | - |
| *sAXL*  *(ng/ml)* | 41 ± 9,  22 – 103 | 43 ± 12,  32 – 63 | 42 ± 11,  24 – 87 | 46 ± 9,  28 – 75 | KW p = 0.005  A+ T+ > A- T- (0.003) | BMI (0.037) | 0.004 |
| sTyro3  (pg/ml) | 1504 ± 348,  547 - 3038 | 1441 ± 559,  1095 - 2768 | 1440 ± 390,  761 - 3017 | 1581 ± 373,  626 - 2406 | KW p = 0.269 | BMI (0.004) | 0.247 |
| CRP  (µg/ml) | 1.5 ± 7.7,  0.2 – 89.9 | 1.5 ± 1.3,  0.7 – 3.8 | 1.3 ± 3.6,  0.2 – 20.0 | 1.3 ± 7.2,  0.1 – 46.8 | KW p = 0.935 | - | - |
| YKL-40  (ng/ml) | 79 ± 114,  23 - 1020 | 99 ± 47,  45 - 178 | 90 ± 386,  20 - 3178 | 115 ± 137,  17 - 810 | KW p = 0.111 | Age (0.001)  Sex (0.001) | 0.664 |
| IL-6  (pg/ml) | 1.6 ± 2.9,  0.5 – 34.0 | 1.6 ± 3.9,  0.9 – 11.7 | 1.9 ± 3.0,  0.4 – 19.8 | 1.8 ± 2.0,  0.7 – 11.3 | KW p = 0.055 | Age (3x10E-5)  BMI (0.013) | 0.704 |
| Il-18  (pg/ml) | 248 ± 132,  113 – 948 | 247 ± 144,  126 – 510 | 240 ± 108,  125 – 781 | 249 ± 186,  84 – 1222 | KW p = 0.971 | Sex (1x10E-7) | 0.769 |
| CXCL10  (pg/ml) | 343 ± 198,  134 – 1312 | 304 ± 223,  97 – 815 | 388 ± 1154,  110 – 9973 | 368 ± 154,  152 – 768 | KW p = 0.293 | Age (0.008) | 0.154 |
| CCL2  (pg/ml) | 343 ± 91,  182 - 662 | 345 ± 96,  247 - 533 | 366 ± 194,  218 – 1729 | 333 ± 97,  218 - 635 | KW p = 0.354 | - | - |
| MIF  (ng/ml) | 16.8 ± 17.2,  7.6 – 196.0 | 19.1 ± 19.0,  9.6 – 66.3 | 16.8 ± 12.3,  10.0 – 103.4 | 15.4 ± 12.5,  7.1 – 74.9 | KW p = 0.184 | - | - |
| C1q  (µg/ml) | 133 ± 25,  85 - 247 | 130 ± 23,  94 - 156 | 130 ± 43,  60 – 372 | 142 ± 37,  81 - 348 | KW p = 0.267 | APOE (0.038) | 0.626 |
| C3  (µg/ml) | 25.4 ± 37.7,  9.6 – 365.6 | 26.1 ± 6.3,  20.0 – 38.9 | 27.5 ± 34.9,  8.2 – 256.0 | 25.3 ± 17.2,  9.9 – 80.2 | KW p = 0.564 | BMI (0.041) | 0.615 |
| C3b  (µg/ml) | 203 ± 138,  43 - 1413 | 143 ± 88,  121 - 368 | 192 ± 281,  114 - 2164 | 186 ± 198,  59 - 1549 | KW p = 0.053 | - | - |
| C4  (µg/ml) | 124 ± 21,  84 – 189 | 115 ± 25,  93 – 156 | 125 ± 47,  84 – 478 | 131 ± 36,  60 – 337 | KW p = 0.353 | Sex (0.016)  BMI (0.013) | 0.121 |
| Factor B  (µg/ml) | 251 ± 45,  149 - 373 | 262 ± 77,  171 - 386 | 236 ± 49,  160 - 404 | 240 ± 57,  145 - 482 | KW p = 0.165 | Sex (0.019)  BMI (0.007) | 0.443 |
| Factor H  (µg/ml) | 363 ± 56,  227 – 528 | 356 ± 65,  274 – 446 | 348 ± 64,  218 – 558 | 359 ± 59,  232 – 635 | KW p = 0.508 | Sex (0.020)  BMI (1x10E-8) | 0.840 |
| FABP-3  (pg/ml) | 5033 ± 2263,  1735 - 26560 | 5078 ± 1903,  2969 - 8932 | 5609 ± 3472,  2449 - 23078 | 6017 ± 2977,  3156 - 18262 | KW p = 0.001  None after adjustment | Age (5x10E-5)  Sex (1x10E-5) | 0.114 |
| Neurogranin  (pg/ml) | 249 ± 319,  77 - 3789 | 229 ± 749,  130 - 2231 | 268 ± 245,  87 - 1533 | 230 ± 187,  35 - 1337 | KW p = 0.162 | - | - |
| Ferritin  (ng/ml) | 314 ± 216,  20 – 992 | 221 ± 245,  31 – 616 | 304 ± 222,  17 – 1031 | 265 ± 222,  26 – 972 | KW p = 0.709 | Sex (1x10E-4)  BMI (0.001) | 0.250 |
| ApoE  (ng/ml) | 6061 ± 3505,  1736 – 3510 | 5885 ± 2437,  4915 – 12000 | 4811 ± 2539,  1124 – 15432 | 4321 ± 2385,  1402 – 17673 | 8x10E-8  None after adjustment | Sex (2x10E-4)  APOE (5x10E-10) | 0.673 |

**Additional Table 4: Biomarkers by A/N scheme**

Experimental biomarkers between A/N (Amyloid / t-tau) scheme groups of subjects by median ± standard deviation, minimum and maximum value. Differences between subject groups were tested by non-parametric Kruskal-Wallis test, followed by ANCOVA using log-transformed protein level values to approximate normal distribution. In ANCOVA, first sensitivity assessment for covariates was performed including all 4 covariates (age, sex, *APOE* status, BMI), followed by a final model including only the significant covariates. Covariates are reported with their ANCOVA significance test result. ANCOVA p is reported for the covariate adjusted group comparison. Markers are highlighted *italic* if they reached α = 0.05 (sAXL, neurogranin), and **bold** if they reached Bonferroni-adjusted α = 0.0025 (not reached by any marker after adjustment for covariates). For pairwise comparisons of single groups, all p values are adjusted for multiple testing with α set at 0.05.

| **Marker** | **A- N-** | **A- N+** | **A+ N-** | **A+ N+** | **Kruskal-**  **Wallis** | **Covariates** | **ANCOVA p** |
| --- | --- | --- | --- | --- | --- | --- | --- |
| N | 153 | 20 | 51 | 83 |  |  |  |
|  |  |  |  |  |  |  |  |
| sTREM2  (ng/ml) | 7.6 ± 9.9,  1.6 – 69.4 | 10.2 ± 19.8,  2.2 – 76.0 | 8.7 ± 23.3,  2.2 – 140.2 | 8.3 ± 16.5,  2.0 – 72.8 | KW p = 0.749 | - | - |
| *sAXL*  *(ng/ml)* | 41.1 ± 9.3,  22.1 – 103.1 | 39.8 ± 10.0,  27.2 – 62.8 | 42.4 ± 9.6,  23.7 – 82.5 | 44.3 ± 10.5,  23.8 – 87.3 | KW p = 0.006  A+ N+ > A- N- (0.009) | BMI (0.037) | 0.004 |
| sTyro3  (pg/ml) | 1498 ± 349,  547 - 3038 | 1510 ± 421,  885 - 2768 | 1509 ± 375,  761 - 3017 | 1542 ± 391,  626 - 2488 | KW p = 0.983 | BMI (0.005) | 0.839 |
| CRP  (µg/ml) | 1.5 ± 7.9,  0.2 – 89.9 | 1.7 ± 2.3,  0.3 – 10.1 | 1.8 ± 3.9,  0.2 – 20.0 | 1.1 ± 6.4,  0.1 – 46.8 | KW p = 0.430 | *APOE* (0.010)  BMI (0.001) | 0.505 |
| YKL-40  (ng/ml) | 79 ± 113,  23 - 1020 | 103 ± 106,  25 - 403 | 94 ± 150,  27 - 976 | 99 ± 356,  17 - 3178 | KW p = 0.130 | Age (0.001)  BMI (0.003) | 0.483 |
| IL-6  (pg/ml) | 1.6 ± 3.0,  0.6 – 34.0 | 1.6 ± 2.4,  0.5 – 11.7 | 2.0 ± 2.5,  0.4 – 11.7 | 1.8 ± 2.6,  0.7 – 19.8 | KW p = 0.057 | Age (3x10E-8) | 0.123 |
| Il-18  (pg/ml) | 245 ± 134,  113 – 948 | 266 ± 122,  126 – 598 | 256 ± 91,  128 – 513 | 248 ± 176,  84 – 1222 | KW p = 0.674 | Sex (9x10E-8) | 0.429 |
| CXCL10  (pg/ml) | 341 ± 201,  134 – 1312 | 349 ± 180,  97 – 829 | 398 ± 253,  110 – 1557 | 368 ± 1063,  152 – 9973 | KW p = 0.267 | Age (0.013) | 0.513 |
| CCL2  (pg/ml) | 346 ± 88,  182 - 634 | 319 ± 114,  220 - 662 | 377 ± 109,  221 – 797 | 343 ± 181,  218 - 1729 | KW p = 0.256 | - | - |
| MIF  (ng/ml) | 16.8 ± 16.7,  7.6 – 196.4 | 18.7 ± 20.8,  9.6 – 97.4 | 16.8 ± 7.2,  10.0 – 53.2 | 16.0 ± 14.7,  7.1 – 103.4 | KW p = 0.259 | - | - |
| C1q  (µg/ml) | 133 ± 25,  85 - 247 | 126 ± 27,  85 - 181 | 135 ± 49,  60 – 372 | 135 ± 35,  81 - 348 | KW p = 0.471 | - | - |
| C3  (µg/ml) | 25.9 ± 39.0,  9.6 – 365.6 | 23.1 ± 6.7,  13.5 – 38.9 | 29.6 ± 39.8,  8.2 – 256.0 | 25.0 ± 16.2,  9.9 – 80.2 | KW p = 0.024 | Sex (0.020)  BMI (0.020) | 0.090 |
| C3b  (µg/ml) | 202 ± 142,  43 - 1413 | 171 ± 89,  105 - 368 | 189 ± 326,  105 - 2164 | 191 ± 176,  59 - 1549 | KW p = 0.395 | - | - |
| C4  (µg/ml) | 124 ± 22,  84 – 189 | 121 ± 21,  93 – 158 | 126 ± 23,  91 – 195 | 128 ± 50,  60 – 478 | KW p = 0.447 | Sex (0.037) | 0.211 |
| Factor B  (µg/ml) | 251 ± 45,  149 - 373 | 261 ± 57,  171 - 386 | 236 ± 55,  160 - 404 | 239 ± 52,  145 - 482 | KW p = 0.184 | Sex (0.013)  BMI (0.002) | 0.341 |
| Factor H  (µg/ml) | 362 ± 55,  227 – 528 | 382 ± 62,  274 – 451 | 338 ± 69,  218 – 558 | 358 ± 57,  232 – 635 | KW p = 0.589 | Sex (0.019)  BMI (8x10E-9) | 0.781 |
| FABP-3  (pg/ml) | 4996 ± 2316,  1735 - 26560 | 5242 ± 1636,  2969 - 8932 | 5609 ± 3070,  2449 - 17753 | 6039 ± 3350,  2694 - 23078 | KW p = 0.001  None after adjustment | Age (6x10E-5)  Sex (2x10E-5) | 0.109 |
| *Neurogranin*  (pg/ml) | 247 ± 173,  77 - 1219 | 253 ± 888,  130 - 3789 | 290 ± 267,  127 - 1533 | 230 ± 182,  35 - 1337 | KW p = 0.032  A+ N+ < A+ N- (0.019) | - | - |
| Ferritin  (ng/ml) | 319 ± 211,  20 – 992 | 176 ± 257,  31 – 720 | 321 ± 203,  38 – 849 | 260 ± 232,  17 – 1031 | KW p = 0.275 | Sex (2x10E-4)  BMI (0.002) | 0.194 |
| ApoE  (ng/ml) | 5946 ± 3603,  1736 – 35094 | 6396 ± 2150,  4123 – 12000 | 4868 ± 2705,  1670 – 15432 | 4371 ± 2309,  1124 – 17673 | KW p = 4x10E-8  None after adjustment | Sex (2x10E-4)  APOE (2x10E-19) | 0.700 |

**Additional Table 5: Biomarkers by AD Spectrum scheme**

Experimental biomarkers between AD spectrum scheme groups of subjects (HC A- T- Versus AD Spectrum: SCD, MCI and DAT A+T- and A+T+) by median ± standard deviation, minimum and maximum value. Differences between subject groups were tested by non-parametric Mann-Whitney U test, followed by ANCOVA using log-transformed protein level values to approximate normal distribution. In ANCOVA, first sensitivity assessment for covariates was performed including all 4 covariates (age, sex, *APOE* status, BMI), followed by a final model including only the significant covariates. Covariates are reported with their ANCOVA significance test result. ANCOVA p is reported for the covariate adjusted group comparison. Markers are highlighted *italic* if they reached α = 0.05 (sAXL, MIF, C3b), and **bold** if they reached Bonferroni-adjusted α = 0.0025 (not reached by any marker) after adjustment for covariates. For pairwise comparisons of single groups, all p values are adjusted for multiple testing with α set at 0.05.

| **Marker** | **HC A-T-** | **AD Spectrum** | **Mann-Whitney U** | **Covariates** | **ANCOVA p** |
| --- | --- | --- | --- | --- | --- |
| N | 55 | 114 |  |  |  |
|  |  |  |  |  |  |
| sTREM2  (ng/ml) | 9.1 ± 5.9,  1.6 – 24.9 | 16.6 ± 20.5,  2.0 – 140.2 | MW p = 0.071 | Age (1x10E-4) | 0.827 |
| *sAXL*  (ng/ml) | 41.3 ± 8.2,  22.1 – 65.7 | 46.3 ± 10.6,  23.7 – 87.3 | MW p = 0.005 | - | - |
| sTyro3  (pg/ml) | 1511 ± 412,  547 - 3038 | 1501 ± 405,  626 - 3017 | KW p = 0.876 | - | - |
| CRP  (µg/ml) | 3.5 ± 5.0,  0.3 – 25.0 | 3.3 ± 5.9,  0.1 – 46.8 | MW p = 0.244 | BMI (0.003) | 0.271 |
| YKL-40  (ng/ml) | 135 ± 162,  23 - 102 | 171 ± 317,  17 - 3178 | MW p = 0.093 | Age (0.003)  Sex (0.018) | 0.552 |
| IL-6  (pg/ml) | 2.5 ± 4.5,  0.7 – 33.7 | 2.9 ± 2.5,  0.7 – 19.8 | MW p = 0.015 | Age (0.001) | 0.698 |
| Il-18  (pg/ml) | 274 ± 128,  113 – 948 | 283 ± 156,  84 – 1222 | MW p = 0.925 | Sex (0.020) | 0.737 |
| CXCL10  (pg/ml) | 407 ± 240,  134 – 1312 | 480 ± 915,  110 – 9973 | MW p = 0.528 | Age (0.018) | 0.438 |
| CCL2  (pg/ml) | 364 ± 100,  213 – 662 | 367 ± 163,  218 – 1729 | MW p = 0.688 | - | - |
| *MIF*  (ng/ml) | 20.4 ± 12.4,  11.2 – 97.3 | 19.2 ± 13.1,  7.1 – 103.4 | MW p = 0.036 | - | - |
| C1q  (µg/ml) | 141 ± 23,  97 – 187 | 143 ± 42,  60 - 372 | MW p = 0.805 | - | - |
| C3  (µg/ml) | 31.5 ± 18.1,  9.6 – 103.1 | 35.6 ± 31.3,  8.2 – 256.0 | MW p = 0.742 | - | - |
| *C3b*  (µg/ml) | 237 ± 82,  81 - 531 | 242 ± 264,  59 – 2164 | MW p = 0.018 | - | - |
| C4  (µg/ml) | 129 ± 21,  90 – 182 | 136 ± 44,  85 – 478 | MW p = 0.529 | - | - |
| Factor B  (µg/ml) | 250 ± 47,  170 – 366 | 247 ± 52,  166 – 482 | MW p = 0.417 | - | - |
| Factor H  (µg/ml) | 362 ± 56,  227 – 458 | 357 ± 60,  224 – 635 | MW p = 0.219 | BMI (0.001) | 0.981 |
| FABP-3  (pg/ml) | 5157 ± 1504,  1763 - 8868 | 6752 ± 3308,  2449 - 23078 | MW p = 0.001 | Age (1x10E-4)  Sex (0.004) | 0.309 |
| Neurogranin  (pg/ml) | 370 ± 503,  77 - 3789 | 291 ± 203,  35 - 1533 | MW p = 0.390 | - | - |
| Ferritin  (ng/ml) | 370 ± 222,  20 – 992 | 329 ± 228,  17 – 1031 | MW p = 0.186 | Sex (0.002)  BMI (1x10E-4) | 0.054 |
| ApoE  (ng/ml) | 6919 ± 3457,  2185 – 20534 | 4751 ± 2344,  1124 – 17673 | MW p = 1x10E-6 | Sex (8x10E-5)  Age (0.014)  *APOE* (2x10E-13) | 0.087 |

|  |  |  |  |  |  |  |  |
| --- | --- | --- | --- | --- | --- | --- | --- |

**Additional Figure 2: Braak ROI alternate models**

Correlation matrix between Braak stage ROI scores and biomarker panel. **A)** Matrix adjusted against the covariates age, sex, BMI and *APOE* status, excluding the MCI and AD subjects. The previously consistent correlations for multiple serum markers against Braak ROI I, for YKL-40 and IL-6 turned insignificant in this model. **B)** Matrix with additional adjustment of serum relations against Aβ42/40 and p-tau-181. Results were mostly identical to those without adjustment for AD biomarker level. Unadjusted CSF correlations are provided for effect comparison.


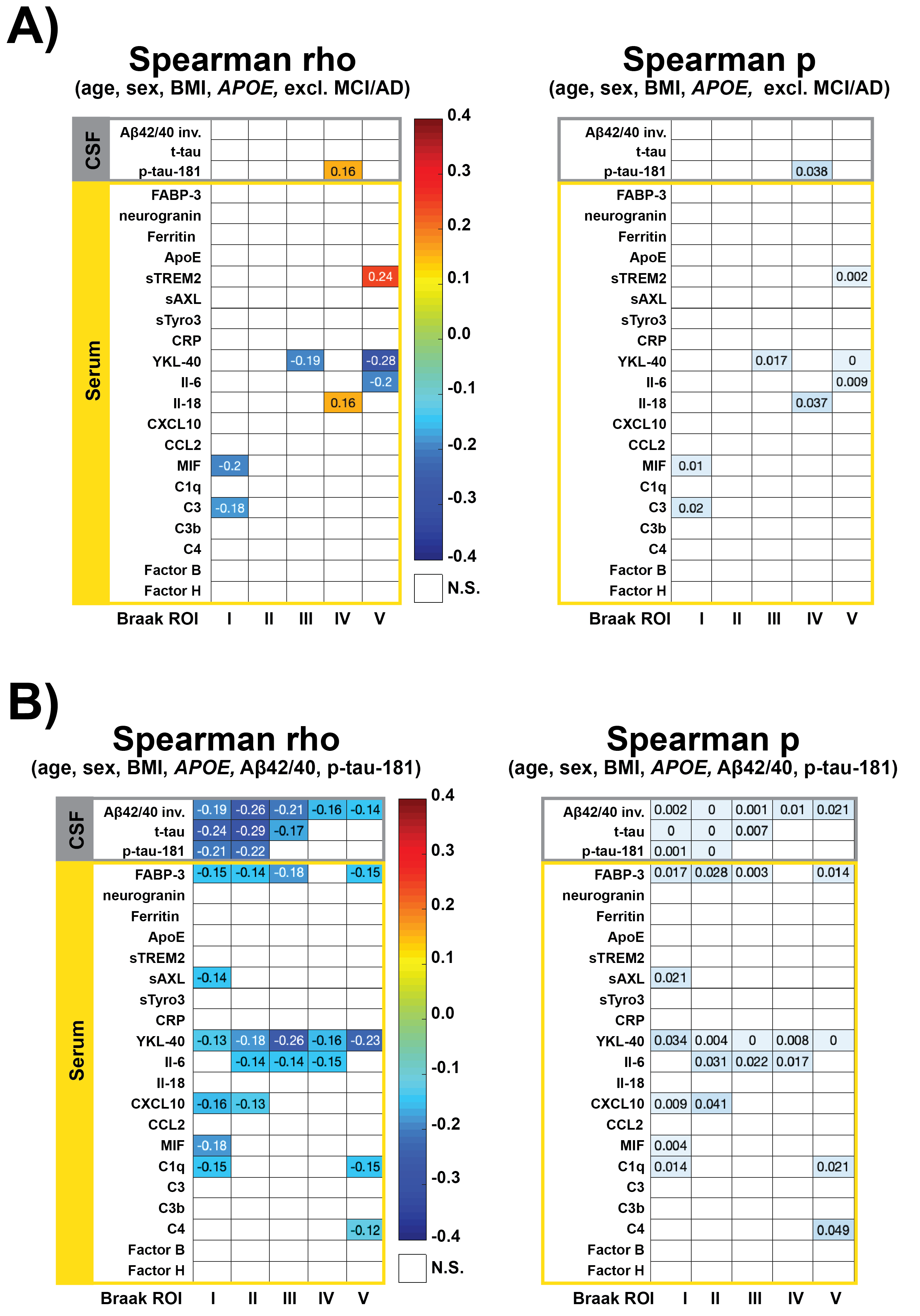


**Additional Table 6: PACC5 Score analysis**

Relation of AD-biomarker associated inflammatory markers with cognitive function at baseline and cognitive decline over up to five years using latent process mixed models adjusted for age, sex, BMI and *APOE* status. The table displays results from calculation performed on the whole cohort with available PACC score data (baseline N = 289, total of 1011 longitudinal observations): Est, effect estimate; SE, standard error of estimate, t-value, p-value of significance test. Markers with significant test results (p < 0.05) at baseline or follow-up are highlighted **bold**.

| **Marker** | **PACC5 at baseline**  **(main effect of marker)** | | | | **Change in PACC5**  **(marker*time interaction)** | | | |
| --- | --- | --- | --- | --- | --- | --- | --- | --- |
|  | Est | SE | t | p | Est | SE | t | p |
| sTREM2 | -0.30 | 0.16 | -1.88 | 0.060 | -0.01 | 0.039 | -0.36 | 0.722 |
| **sAXL** | **-0.48** | **0.16** | **-2.97** | **0.003** | -0.06 | 0.04 | -1.58 | 0.114 |
| sTyro3 | -0.20 | 0.18 | -1.15 | 0.249 | 0.01 | 0.03 | 0.43 | 0.669 |
| CRP | -0.25 | 0.17 | -1.46 | 0.145 | -0.05 | 0.03 | -1.69 | 0.090 |
| YKL-40 | -0.21 | 0.14 | -1.50 | 0.134 | 0.01 | 0.04 | 0.22 | 0.827 |
| IL-6 | 0.18 | 0.19 | 1.00 | 0.320 | 0.03 | 0.03 | 0.88 | 0.377 |
| Il-18 | -0.32 | 0.17 | -1.87 | 0.061 | 0.07 | 0.04 | 1.92 | 0.055 |
| CXCL10 | -0.05 | 0.13 | -0.35 | 0.728 | 0.08 | 0.04 | 1.88 | 0.061 |
| CCL2 | -0.02 | 0.18 | -0.14 | 0.891 | 0.04 | 0.03 | 1.59 | 0.111 |
| MIF | 0.10 | 0.18 | 0.54 | 0.587 | -0.04 | 0.03 | -1.28 | 0.201 |
| C1q | -0.10 | 0.16 | -0.62 | 0.535 | 0.03 | 0.04 | 0.70 | 0.486 |
| C3 | -0.01 | 0.19 | -0.03 | 0.977 | 0.02 | 0.03 | -0.47 | 0.636 |
| C3b | -0.06 | 0.17 | -0.33 | 0.742 | 0.01 | 0.04 | 0.18 | 0.855 |
| C4 | -0.27 | 0.14 | -1.92 | 0.055 | -0.04 | 0.03 | -1.04 | 0.296 |
| Factor B | -0.21 | 0.17 | -1.20 | 0.231 | -0.04 | 0.03 | -1.20 | 0.231 |
| Factor H | -0.19 | 0.17 | -1.07 | 0.287 | 0.01 | 0.03 | 0.344 | 0.731 |
| **FABP-3** | -0.17 | 0.16 | -1.04 | 0.301 | **-0.12** | **0.04** | **-2.97** | **0.003** |
| Neurogranin | 0.30 | 0.17 | 1.77 | 0.076 | -0.02 | 0.03 | -0.64 | 0.520 |
| Ferritin | 0.22 | 0.18 | 1.26 | 0.210 | 0.02 | 0.03 | 0.70 | 0.485 |
| **ApoE** | **-0.38** | **0.18** | **-2.07** | **0.039** | -0.01 | 0.04 | -0.14 | 0.889 |

**Additional Table 7: PACC5 Score alternate models**

PACC was modelled for sAXL, FABP-3 and ApoE adjusted for age, sex, BMI and *APOE* status as described in supplementary table 5. Est, effect estimate; SE, standard error of estimate, t-value, p-value of significance test. Markers with significant test results (p < 0.05) at baseline or follow-up are highlighted **bold**. Model A: Additional adjustment against CSF Aβ42/40 and p-tau-181. Model B: No additional adjustment, but exclusion of MCI and AD subjects from the analysis. Model A results resembled the model without additional adjustment for Aβ42/40 and p-tau-181, whereas exclusion of MCI and AD subjects in model B lead to loss of significant effects.

| **Marker** | **Model** | **PACC5 at baseline**  **(main effect of marker)** | | | | **Change in PACC5**  **(marker*time interaction)** | | | |
| --- | --- | --- | --- | --- | --- | --- | --- | --- | --- |
|  |  | Est | SE | t | p | Est | SE | t | p |
| sAXL | A | **-0.41** | **0.15** | **-2.66** | **0.008** | -0.05 | 0.04 | -1.28 | 0.199 |
|  | B | -0.07 | 0.15 | -0.43 | 0.669 | 0.03 | 0.04 | 0.91 | 0.364 |
| FABP-3 | A | -0.07 | 0.15 | -0.43 | 0.667 | **-0.11** | **0.04** | **-2.67** | **0.008** |
|  | B | 0.08 | 0.16 | 0.52 | 0.606 | -0.08 | 0.05 | -1.66 | 0.097 |
| ApoE | A | **-0.45** | **0.17** | **-2.64** | **0.008** | -0.01 | 0,04 | -0.14 | 0.893 |
|  | B | -0.07 | 0.18 | -0.41 | 0.686 | 0.03 | 0.04 | 0.60 | 0.550 |

**Additional Figure 3: Human Protein Atlas: Brain RNA expression**

Heat map of brain RNA expression level data, matching the proteins of the experimental biomarker panel, derived of the Human Protein Atlas (<https://www.proteinatlas.org/>). For each marker, protein and gene name are listed and plotted against normalized transcripts per million protein coding genes (nTPM) counts. Color Coding is based on protein-specific minimum / maximum nTPM count.


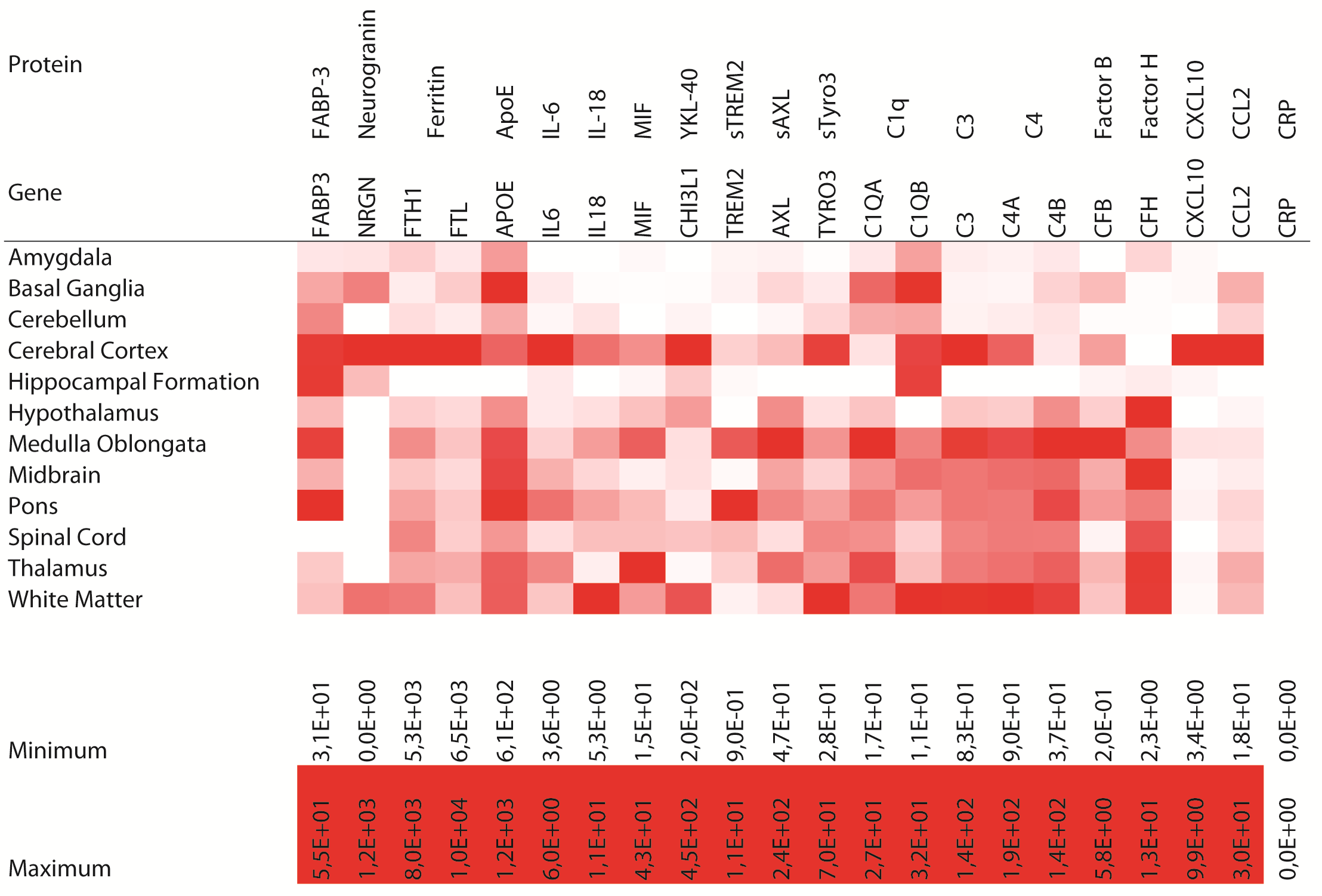


**Additional Figure 4: Human Protein Atlas: Blood RNA expression**

Heat map of blood RNA expression level data, matching the proteins of the experimental biomarker panel, derived of the Human Protein Atlas (<https://www.proteinatlas.org/>). For each marker, protein and gene name are listed and plotted against normalized transcripts per million protein coding genes (nTPM) counts. Color Coding is based on protein-specific minimum / maximum nTPM count.


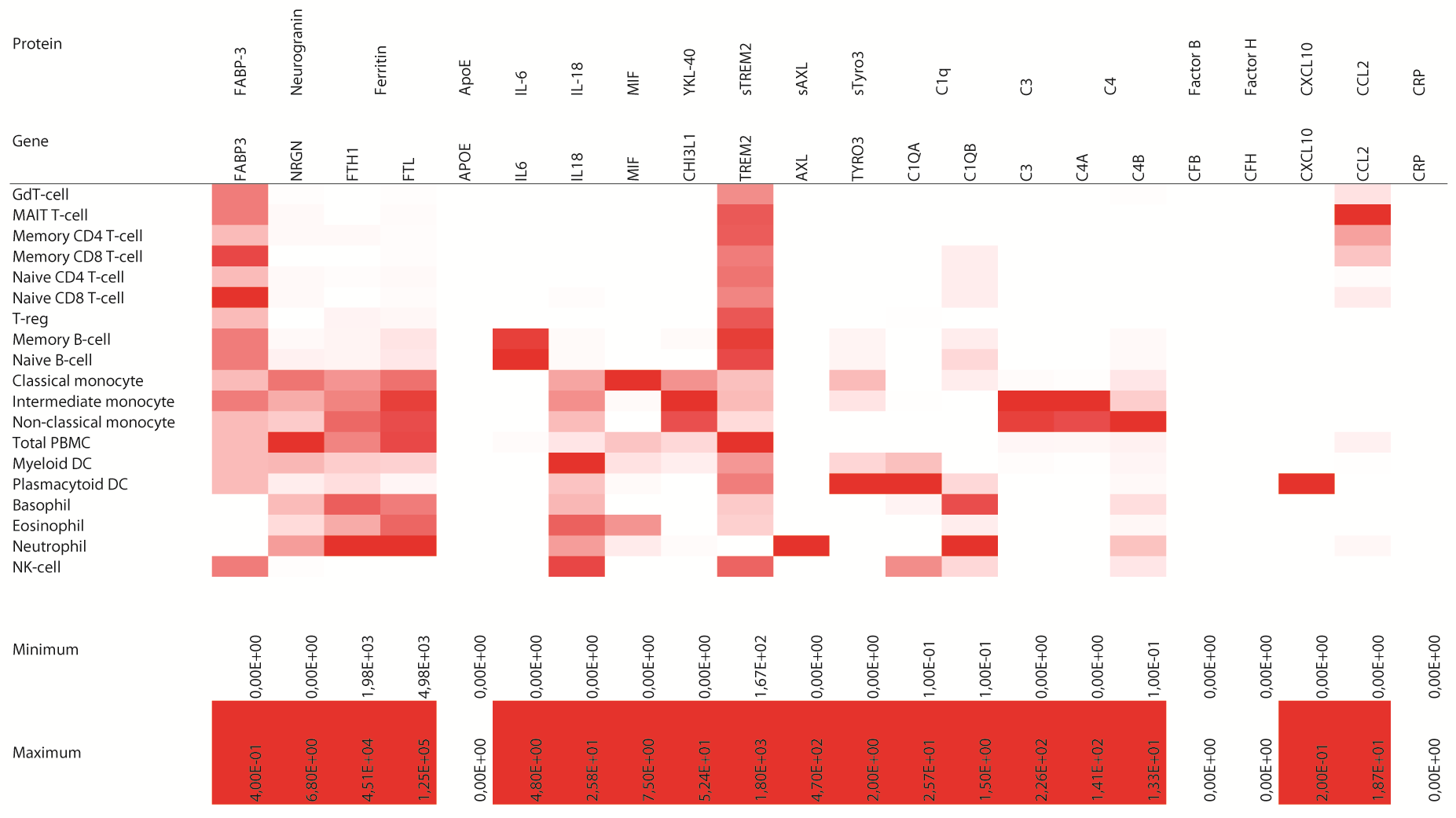


**Additional Figure 5: Human Protein Atlas: Tissue RNA expression**

Heat map of tissue RNA expression level data, matching the proteins of the experimental biomarker panel, derived of the Human Protein Atlas (<https://www.proteinatlas.org/>). For each marker, protein and gene name are listed and plotted against normalized transcripts per million protein coding genes (nTPM) counts. Color Coding is based on protein-specific minimum / maximum nTPM count.


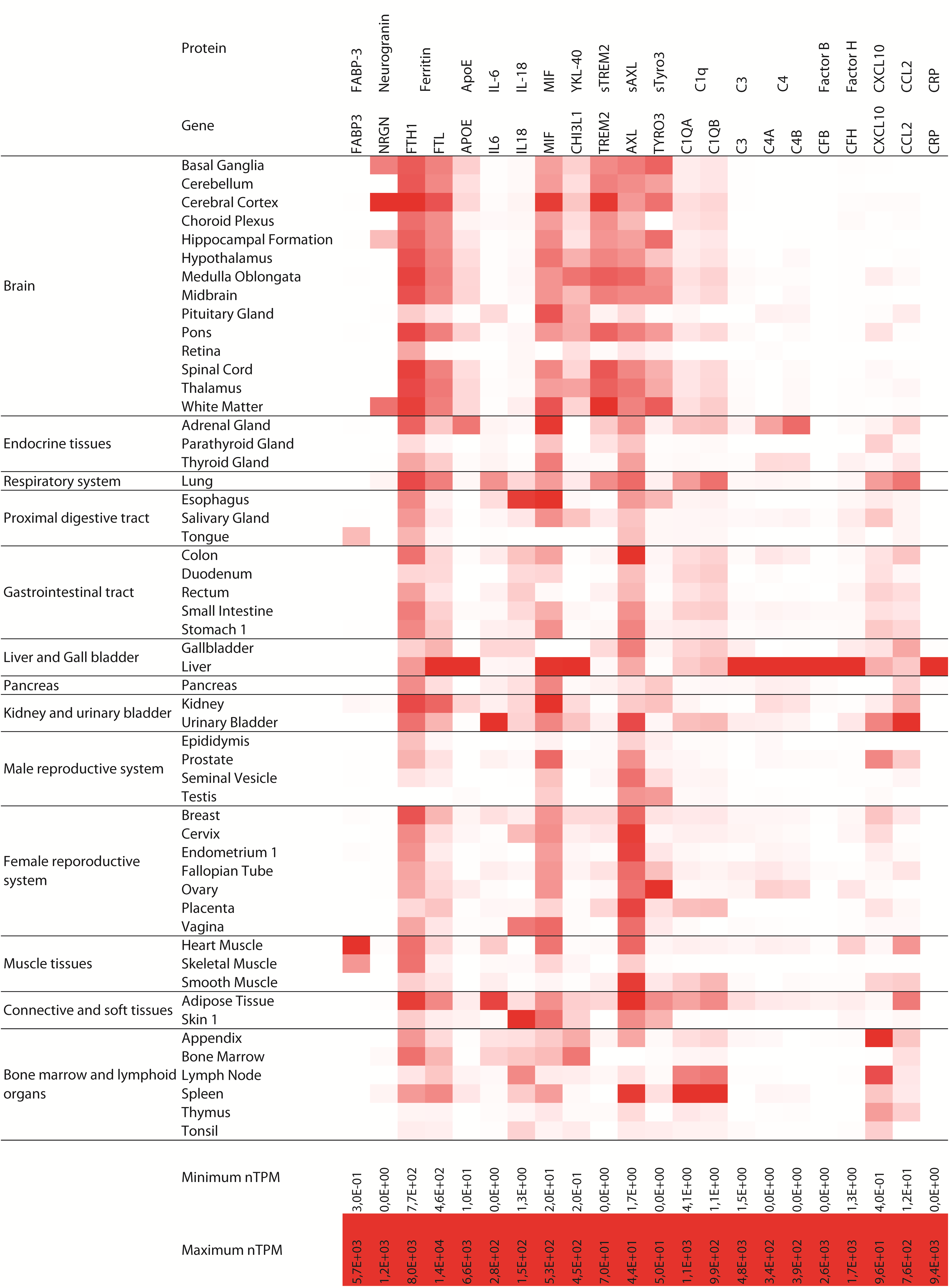


**Additional Figure 6: Human Protein Atlas: Protein Expression Levels**

Heat map of tissue protein expression level data, matching the proteins of the experimental biomarker panel, derived of the Human Protein Atlas (<https://www.proteinatlas.org/>). For each marker, protein and gene name are listed and plotted against tissue protein immunohistochemistry staining annotation, ranked as negative (0), weak (1), moderate (2) or strong (3) as provided by Protein Atlas.


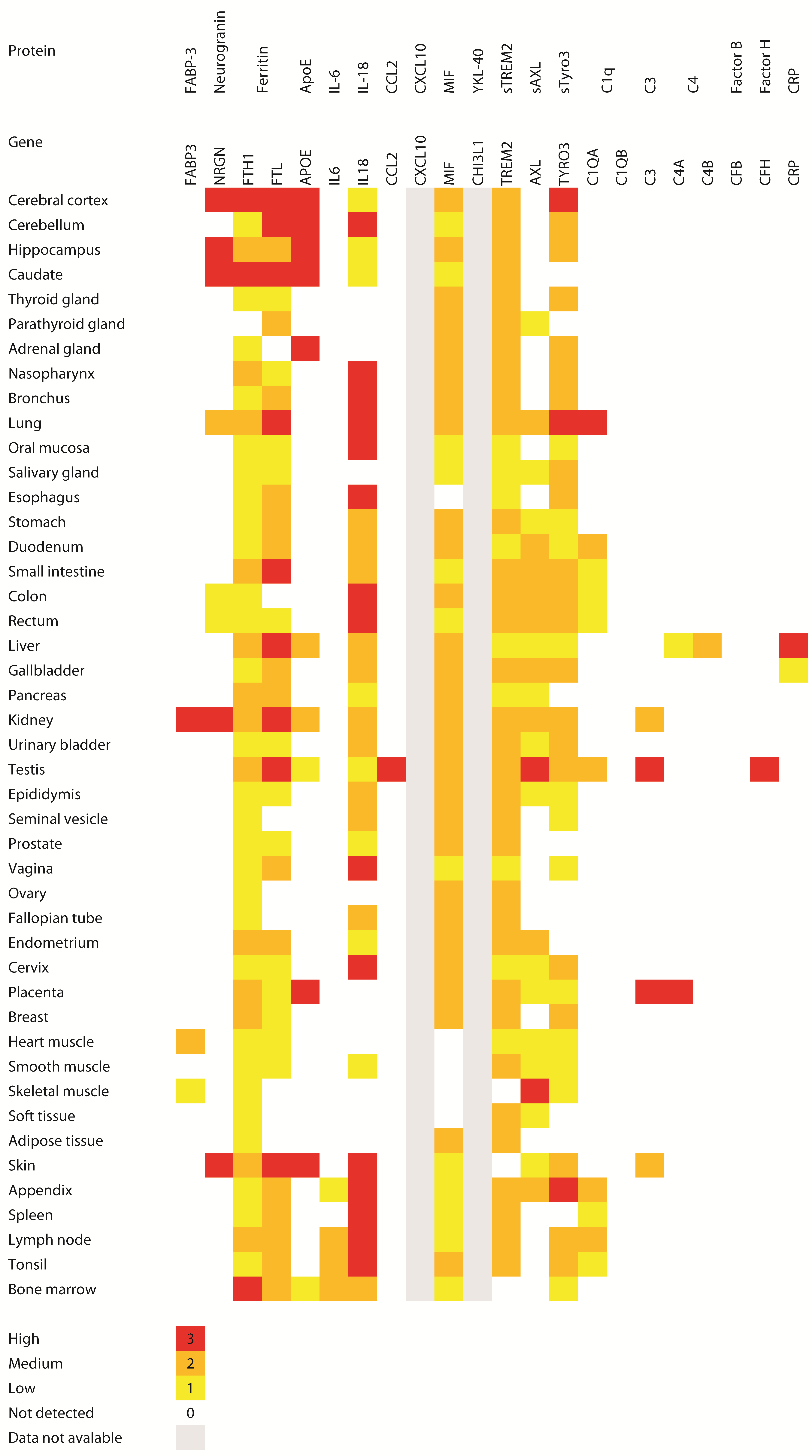

Supplement: Supplementary file 1 — Additional file 1. [file 13195_2022_1118_MOESM1_ESM.doc]
